# Directional cell expansion requires NIMA-related kinase 6 (NEK6)-mediated cortical microtubule destabilization

**DOI:** 10.1038/s41598-017-08453-5

**Published:** 2017-08-10

**Authors:** Shogo Takatani, Shinichiro Ozawa, Noriyoshi Yagi, Takashi Hotta, Takashi Hashimoto, Yuichiro Takahashi, Taku Takahashi, Hiroyasu Motose

**Affiliations:** 1 Department of Biological Science, Graduate School of Natural Science and Technology, Okayama University, 3-1-1 Tsushimanaka, Okayama, 700-8530 Japan; 2 Japan Science and Technology Agency, 4-1-8 Kawaguchi, Saitama, 332-0012 Japan; 3 Graduate School of Biological Science, Nara Institute of Science and Technology, Ikoma, 630-0192 Japan; 4 Department of Applied Biological Science, Tokyo University of Science, Noda, Chiba 278-8510 Japan; 5 Department of Embryology, Carnegie Institution for Science, 3520 San Martin Drive, Baltimore, MD 21218 USA

## Abstract

Plant cortical microtubules align perpendicular to the growth axis to determine the direction of cell growth. However, it remains unclear how plant cells form well-organized cortical microtubule arrays in the absence of a centrosome. In this study, we investigated the functions of *Arabidopsis* NIMA-related kinase 6 (NEK6), which regulates microtubule organization during anisotropic cell expansion. Quantitative analysis of hypocotyl cell growth in the *nek6-1* mutant demonstrated that NEK6 suppresses ectopic outgrowth and promotes cell elongation in different regions of the hypocotyl. Loss of NEK6 function led to excessive microtubule waving and distortion, implying that NEK6 suppresses the aberrant cortical microtubules. Live cell imaging showed that NEK6 localizes to the microtubule lattice and to the shrinking plus and minus ends of microtubules. In agreement with this observation, the induced overexpression of *NEK6* reduced and disorganized cortical microtubules and suppressed cell elongation. Furthermore, we identified five phosphorylation sites in β-tubulin that serve as substrates for NEK6 ***in vitro***. Alanine substitution of the phosphorylation site Thr166 promoted incorporation of mutant β-tubulin into microtubules. Taken together, these results suggest that NEK6 promotes directional cell growth through phosphorylation of β-tubulin and the resulting destabilization of cortical microtubules.

## Introduction

Microtubules are polar cytoskeletal polymers of α/β-tubulin heterodimers that are essential for many cellular processes, such as cell division, morphogenesis, motility, and intracellular trafficking. Tubulin heterodimers polymerize longitudinally in a head-to-tail manner to form a protofilament, and 13 of these protofilaments associate laterally to form a hollow microtubule that is approximately 25 nm in diameter. Microtubules stochastically switch between assembly and disassembly phases, in a process driven by GTP binding and hydrolysis^1^. This property, known as dynamic instability, allows microtubules to probe the intracellular environment and rapidly reorganize in response to changes in this environment. Microtubules are nucleated from a γ-tubulin ring complex (γTuRC). The γTuRC is a lockwasher-like structure with a γ-tubulin symmetry that functions as a template on which microtubules are formed^2^. In animals and fungi, the γTuRC is recruited to the centrosome and spindle pole body, which are microtubule-organizing centers, where they form radial microtubule arrays. Although both the structure and function of microtubules are well conserved, higher plant cells lack distinct microtubule-organizing centers such as a centrosome, but they can form well-organized cortical microtubule arrays that direct cell expansion^3^. Cortical microtubules associate with the inner face of the plasma membrane and align parallel to each other to regulate directional cell expansion and morphogenesis. However, the mechanism underlying the regulation of parallel cortical microtubule arrays in plant cells remains unclear.

Previous studies showed that cortical microtubules possess self-organizing properties. When the growing plus-end of a cortical microtubule encounters an existing microtubule at an angle of less than 40°, the microtubules co-align to form a bundle, and when the angle is greater than 40°, the microtubules undergo catastrophe, which results in the removal of non-parallel microtubules^4^. This angle-dependent organizing mechanism results in a parallel array of cortical microtubules. Cortical microtubules are nucleated from γTuRC on preexisting cortical microtubules to form branches or microtubule bundles. When γTuRC is recruited to a preexisting microtubule, its nucleation activity is stimulated and the microtubule branches off the preexisting microtubule at an angle of approximately 40°, or grows parallel to preexisting microtubules^5–7^. This microtubule-dependent nucleation mechanism establishes the organization of the cortical array.

Microtubule-associated proteins (MAPs) are key regulators of cortical microtubule dynamics and organization. Katanin is a heterodimeric microtubule-severing MAP that consists of p60 catalytic and p80 regulatory subunits^8^. The catalytic subunit of *Arabidopsis* katanin (KTN1) severs newly branched daughter microtubules from the γTuRC^7^. It has been shown that KTN1-mediated microtubule severing at microtubule intersections creates new growing microtubule ends and drives reorientation of cortical microtubules during phototropism^9^. This KTN1-dependent microtubule rearrangement plays a pivotal role in directional cell growth and in shoot apical meristem development^10^. CLIP-associated protein (CLASP) is a plus-end MAP that promotes microtubule polymerization. *Arabidopsis* CLASP has been shown to overcome cell-edge-induced microtubule catastrophe and to regulate microtubule geometry in meristematic cells^11^.

Microtubule organization and dynamics are also regulated by post-translational modification of tubulin. *Arabidopsis* PROPYZAMIDE HYPERSENSITIVE1 (PHS1) has both a MAP kinase phosphatase domain and an atypical kinase domain^12, 13^. The atypical PHS1 kinase domain is activated by osmotic stress and phosphorylates Thr-359 on α-tubulin, which inhibits assembly of tubulin heterodimers into microtubules and ultimately results in cortical microtubule depolymerization^13–15^. *Arabidopsis* Casein kinase 1-like 6 (CKL6) localizes to cortical microtubules and phosphorylates β-tubulin *in vitro* ^16^. An alanine substitution experiment suggested that CKL6 phosphorylates both Ser-413 and Ser-420 at the C-terminus of TUB3 (Ben-Nissan *et al*.^16^), but their function is not known.

NIMA-related kinases (NEKs) are a family of mitotic kinases that are well conserved in most eukaryotes^17^. Fungal and animal NEKs have been shown to regulate various mitotic events, including the G2/M transition, centrosome separation, and spindle formation^17^. These NEK functions are closely associated with the regulation of microtubule function. Specifically, two *Chlamydomonas* NEKs, Fa2p and Cnk2p, are implicated in microtubule severing and deflagellation^18^ and in regulating flagellar length and cell size^19^, respectively. Never in mitosis A (NIMA), the founding member of the NEK family, was discovered in a mitotic mutant, *nimA*, of *Aspergillus nidulans* ^20^. A recent study revealed that NIMA is also required for hyphal tip growth and microtubule organization during interphase in *A*. *nidulans* ^21^.

Although the functions of plant NEKs are largely unknown, previous studies have shown that *Arabidopsis* NEK6 regulates directional cell expansion in interphase^22–26^. A loss-of-function *Arabidopsis nek6* mutant exhibits ectopic protrusions and aberrant cortical microtubule arrays in the epidermal cells of the hypocotyl and petiole, indicating that *NEK6* suppresses ectopic outgrowth by modulating microtubule organization^22–24^. Both the kinase activity and localization to microtubules of NEK6 are required for its function in directional growth^22^. The cortical microtubules of the *nek6* mutant have increased resistance to the microtubule-depolymerizing drug oryzalin^24^. Moreover, the findings that taxol-mediated microtubule stabilization promotes ectopic outgrowth, whereas propyzamide-mediated microtubule depolymerization suppresses it, suggest that NEK6 destabilizes cortical microtubules. In addition, NEK6 interacts with the other NEK members, NEK4 and NEK5, and phosphorylates β-tubulin *in vitro* ^24^. These results imply that NEK6 destabilizes cortical microtubules through phosphorylation of β-tubulin. However, the exact roles of NEK6 in cell elongation and the functional significance of tubulin phosphorylation remain unclear. Here, we quantitatively analyze hypocotyl cell growth of the *nek6–1* mutant (a severe loss-of-function allele) and demonstrate that NEK6 has dual functions in promoting longitudinal cell elongation and suppressing radial ectopic growth. NEK6 preferentially localizes to shrinking ends of microtubules and may regulate depolymerization of cortical microtubules through phosphorylation of β-tubulin. We identified five β-tubulin target sites that undergo NEK6-mediated phosphorylation *in vitro*, and found that Thr166 is the most important phosphorylation site for depolymerization of cortical microtubules when expressed in mutant forms *in vivo*. We propose a novel regulatory mechanism for cortical microtubule arrays in plants in which NEK6 functions to remove aberrant microtubules through phosphorylation of β-tubulin at specific residues.

## Results

### NEK6 promotes cell elongation and suppresses ectopic growth through regulating cortical microtubules

**Figure 1:**
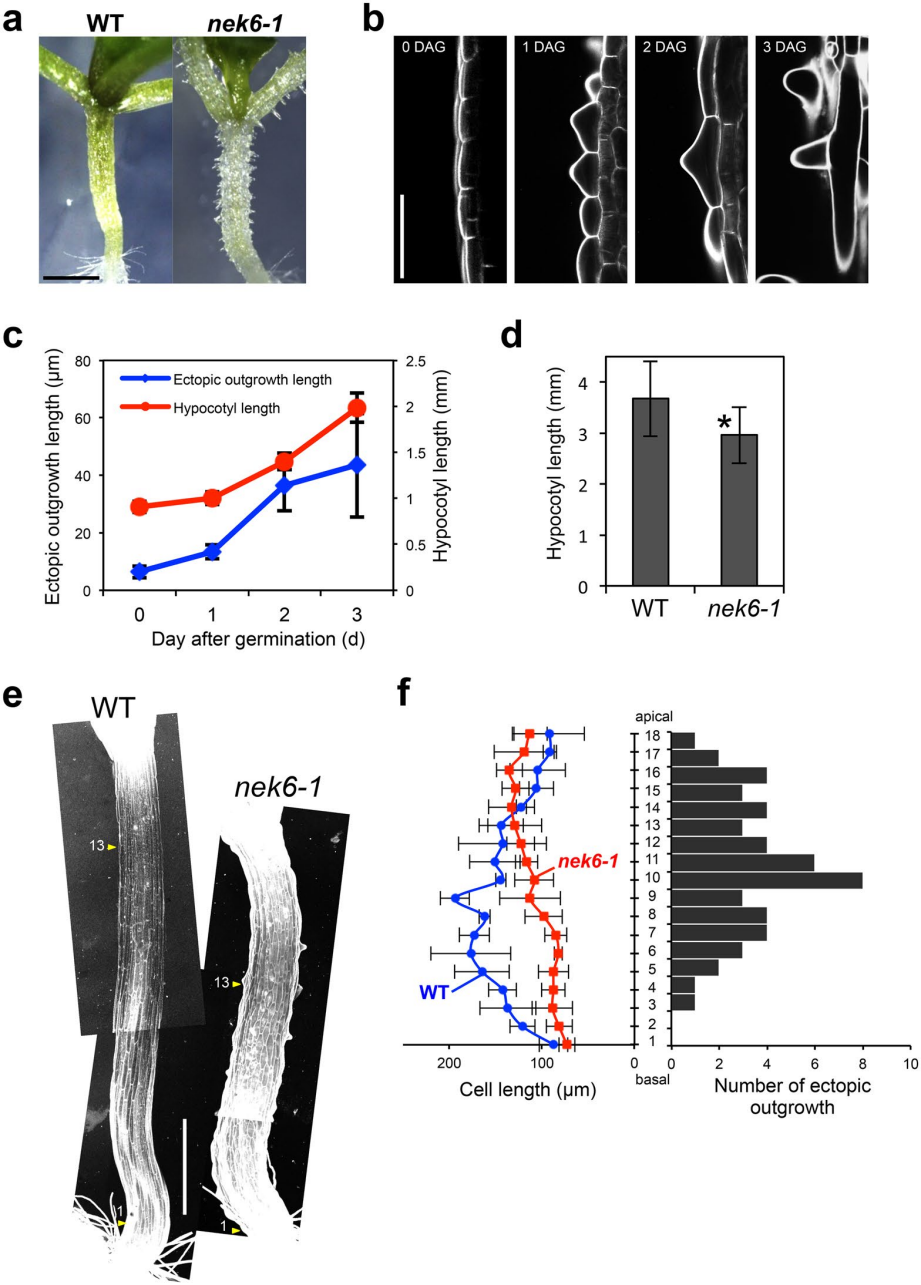
NEK6 suppresses ectopic outgrowth and promotes cell elongation. (**a**) Morphology of wild type (WT) and *nek6-1* mutant seedlings grown for 7 days. The scale bar represents 1 mm. (**b**) Morphology of epidermal cells in the hypocotyls of *nek6-1* seedlings 0 to 3 days after germination (DAG). The seedlings (0 to 3 DAG) were stained with propidium iodide and observed under a confocal microscopy. The scale bar represents 50 µm. (**c**) Quantification of length of ectopic outgrowths and hypocotyls in *nek6-1* seedlings 0 to 3 days after germination (DAG). Data are displayed as averages ± SD (n = 14 to 21 protrusions and n = 10 hypocotyls). (**d**) Quantification of hypocotyl length of wild type (WT) and *nek6-1* seedlings grown for 8 days. Data are displayed as averages ± SD (n = 12). The asterisk indicates significant difference from the value of the wild type (*t*-test, *P* < 0.001). (**e**) Morphology of wild type (WT) and *nek6-1* seedlings 3 days after germination (DAG). The seedlings were stained with propidium iodide and observed under a confocal microscopy. Arrowheads represent the cell number in a file of the hypocotyl with respect to the graph in (**f**) (No. 13 cell from the bottom of the hypocotyl). Scale bar represents 50 µm. (**f**) Quantification of cell length and number of ectopic outgrowth along hypocotyl axes of wild type (WT) and *nek6-1*. The vertical axis indicates cell number from bottom of hypocotyl. The left panel indicates cell length along hypocotyl and data are displayed as averages ± SD (n = 10). The right panel shows a histogram of total number of ectopic protrusions in 10 cell files.

To elucidate the roles of *NEK6* in cell growth, we studied the phenotypes of the *nek6-1* mutant, which exhibits aberrant outgrowths of the epidermal cells of the hypocotyl and petiole (Fig. 1a). Previously, we isolated and characterized a *nek6* mutant named *ibo1-1* in the Wassilewskija (Ws) accession, which showed a relatively mild phenotype^22^. In this study, we investigated the growth dynamics and organization of microtubules in the *nek6-1* mutant, which showed a severe growth defect in the epidermal cells of the hypocotyl and petiole. First, we monitored hypocotyl cell growth over time. Ectopic outgrowths formed on elongating epidermal cells during hypocotyl growth (Fig. 1b,c), implying that ectopic outgrowth is due to a defect in the directional growth of epidermal cells.

In addition to forming ectopic outgrowths, *nek6-1* also showed inhibited hypocotyl growth (Fig. 1d,e). To determine the relationship between ectopic outgrowth and growth suppression, we measured the frequency of ectopic outgrowth formation and the length of epidermal cells at different locations along the hypocotyls (Fig. 1f). Ectopic outgrowths formed predominantly in the middle region of hypocotyls, whereas cell elongation was remarkably suppressed in the basal region of hypocotyls of the *nek6-1* mutant (Fig. 1f). This result suggests that *NEK6* has spatially distinct roles; it suppresses ectopic outgrowth in the middle regions of hypocotyls and promotes cell elongation in the basal regions. These functions might both be required to coordinate the rapid and directional growth of organs, including hypocotyls and petioles.

### NEK6 is required to generate ordered cortical microtubule arrays

**Figure 2:**
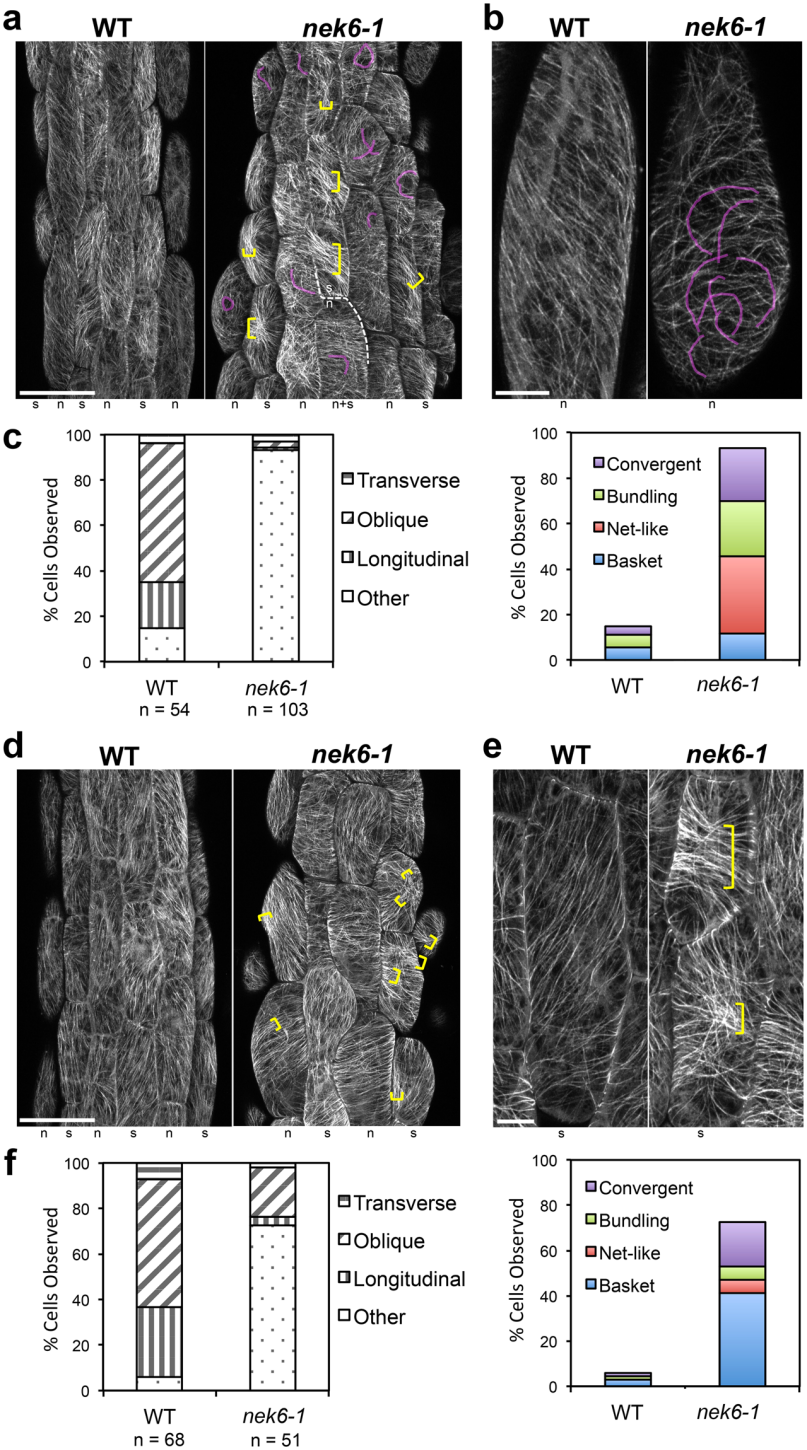
NEK6 regulates cortical microtubule organization during directional cell growth. Cortical microtubule organization visualized by GFP-TUB6 in hypocotyl epidermal cells of 3-day-old seedlings of wild type (WT) and *nek6-1* mutant. (**a**,**b**) Cortical microtubules in the middle region of hypocotyl (No. 7–15 cells from bottom of hypocotyl). The representative cells in the non-stomatal cell files are shown in (**b**). (**c**) Quantification of cortical microtubule arrays in the middle region of hypocotyls. (**d**,**e**) Cortical microtubules in the basal region of hypocotyl (No. 1–6 cells from bottom of hypocotyl). The representative cells in the stomatal cell files are shown in (**e**). (**f**) Quantification of cortical microtubule arrays in the basal region of hypocotyls. In (**a**,**b**) and (**d**,**e**), light magenta lines highlight bended cortical microtubules and yellow brackets indicate microtubule bundling. At the bottom of each panel in (**a**,**b**) and (**d**,**e**), the stomatal and non-stomatal cell file is labeled as “s” and “n”, respectively. In (**c**) and (**f**), the left panels show the percentages of cells having transverse, oblique, or longitudinal arrays. In the right panels, cells with other arrangements are divided into four classes. Scale bars represent 50 µm (**a**,**d**) and 10 µm (**b**,**e**).

To determine whether the dual function of *NEK6* is mediated by microtubules, we examined the organization of cortical microtubules in the epidermal cells of wild-type and *nek6-1* mutant hypocotyls transgenically expressing the microtubule marker GFP-TUB6. In the middle region of *nek6-1* hypocotyls, before ectopic outgrowths formed, cortical microtubules were highly disorganized (Fig. 2a–c). *Arabidopsis* hypocotyls are comprised of stomatal and non-stomatal cell files, and ectopic outgrowth is observed only in the latter^22^. In the non-stomatal cell files, epidermal cells of the wild type exhibited parallel and straight arrays of cortical microtubules, whereas the non-stomatal cell files of the *nek6-1* mutant showed a net-like pattern of microtubules, in which microtubules were frequently bent and intersected with other microtubules (highlighted by the light magenta lines in Fig. 2a,b right panel). In the stomatal cell files of the *nek6-1* mutant, cortical microtubules were organized in bundles (highlighted by the yellow brackets in Fig. 2a). In the basal region of hypocotyls, cortical microtubules of the wild type were arranged in parallel arrays, whereas those of *nek6-1* hypocotyls were bundled and/or exhibited a basket-like organization (Fig. 2d–f). We classified the patterns of cortical microtubules as previously described (transverse, oblique, and longitudinal)^27^ and also subdivided disorganized microtubule patterns without any dominant orientation into four classes: basket, net-like, bundling, and convergent (Fig. S1). This quantitative analysis confirmed disorganization of cortical microtubule arrays in the *nek6-1* mutant (Fig. 2c,f). The net-like pattern was predominant in the middle region of *nek6-1* hypocotyls, whereas the basket pattern was predominant in the basal region (Fig. 2c,f). In summary, net-like microtubule organization was associated with ectopic outgrowth formation, whereas basket-like organization and bundling were associated with the suppression of cell elongation.

### NEK6 is required to suppress distorted cortical microtubules

**Figure 3:**
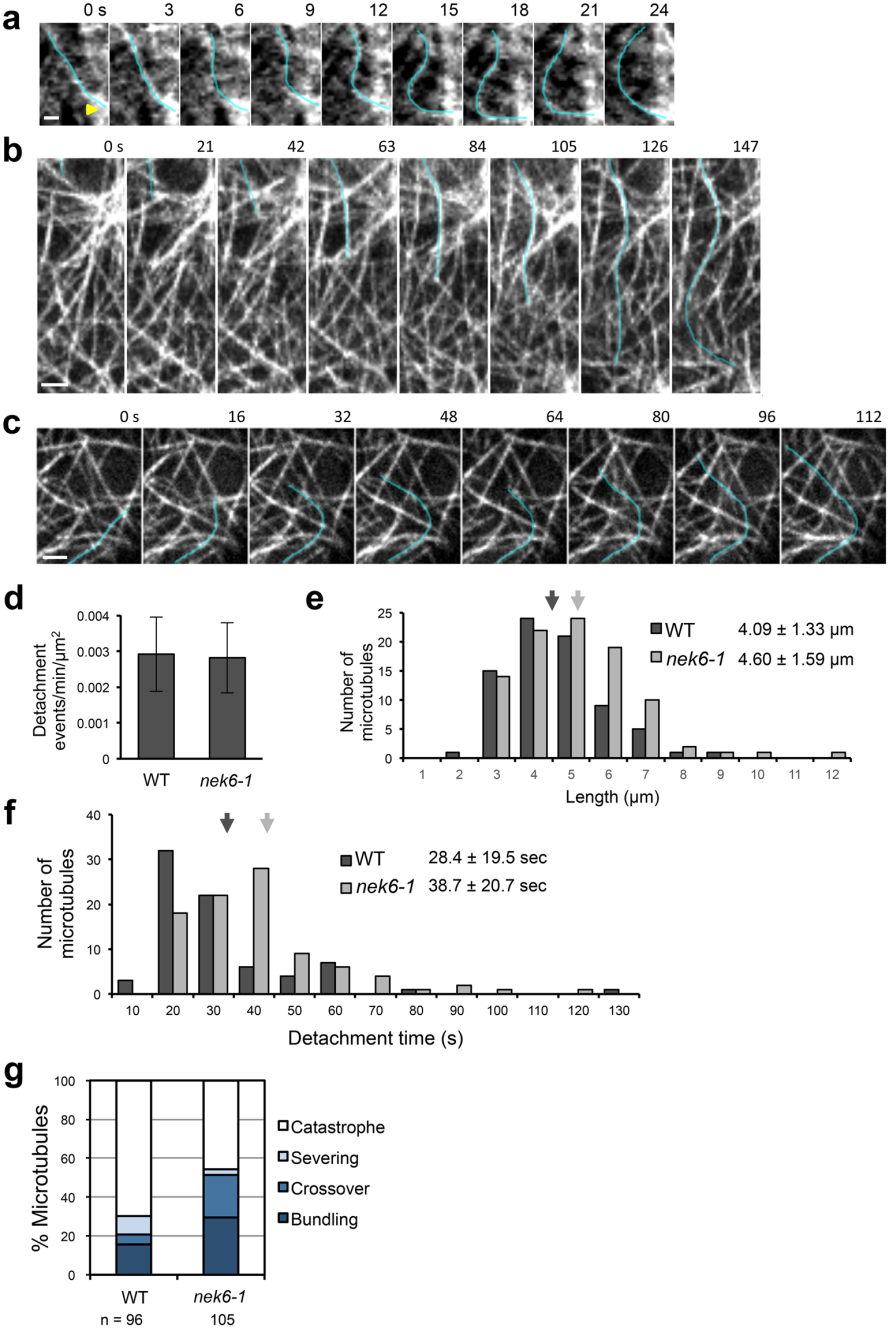
Distorted cortical microtubules in the *nek6-1* mutant. (**a**) A time-lapse montage showing microtubule bending (blue line) near the site of microtubule crossover (arrow) in *nek6-1*. The scale bar represents 1 µm. (**b**) A time-lapse montage showing microtubule distortion (blue line) in a hypocotyl epidermal cell of *nek6-1*. The scale bar represents 2 µm. (**c**) A time-lapse montage showing microtubule distortion (blue line) in a cotyledon epidermal cell of *nek6-1*. The scale bar represents 2 µm. (**d**) Frequency of cortical microtubule detachments in cotyledon epidermal cells of the wild type and *nek6-1*. Data are displayed as averages ± SD. (**e**) Length of detached and distorted cortical microtubules in cotyledon epidermal cells of the wild type and *nek6-1*. Data are displayed as averages ± SD. Arrows indicate means that are significantly different (*t*-test, *P* < 0.03). (**f**) Detachment times of cortical microtubules in cotyledon epidermal cells of the wild type and *nek6-1*. Data are displayed as averages ± SD. Arrows indicate means that are significantly different (*t*-test, *P* < 0.002). (**g**) The fate of detached microtubules in the wild type and *nek6-1*. Microtubules were classified into four categories according to their behaviors after detachment from the cortex: catastrophe, severing, crossover, and bundling.

To determine the origin of the bent microtubules in the *nek6-1* mutant, we analyzed time-lapse images of cortical microtubules. The *nek6-1* mutant had more crossover sites, at which multiple microtubules intersected, than did the wild type, and some of these microtubules were wavy and bent (Figs 2b and 3a and Movies S1–S3). This result suggests that cortical microtubules are trapped at the crossover sites and then exhibit abnormal behavior, such as waving and bending.

The growing ends of cortical microtubules occasionally dissociate from the cell cortex, move laterally, and reorient their growth direction, resulting in microtubule waving and buckling^11, 28^. Thus, we monitored microtubule detachments and buckling in epidermal cells of hypocotyls and cotyledons. In the *nek6-1* mutant, large microtubules were extensively waved and distorted over long times after detaching from the cortex (Fig. 3b,c and Movies S4 and S5). Next, we quantified frequency of dissociation events, length of detached wavy microtubules, and detachment time of cortical microtubules (Fig. 3d–f). The length of detached microtubules and detachment time were significantly increased in the *nek6-1* mutant compared to the wild type (Fig. 3e,f), whereas frequency of dissociation events was same as that in the wild type (Fig. 3d). Furthermore, we monitored the fate of detached microtubules and found that they were classified into four categories: catastrophe (detached microtubules switched from growth to shrinkage and then depolymerized), severing (detached microtubules encountering with preexisting microtubules were severed at microtubule intersections and then depolymerized), crossover (detached microtubules crossed over preexisting microtubules, attached to the cell cortex, and then continued growing), and bundling (detached microtubules encountered with preexisting microtubules, attached to the cell cortex, became bundled with preexisting microtubules and continued growing) (Fig. 3g). The *nek6-1* mutant showed reduction in the percentage of shrinking microtubules detached from the cortex (Fig. 3g). To determine whether NEK6 is required for depolymerization of detached microtubules, we measured catastrophe frequency of detached microtubules in the wild type and *nek6-1* mutant. The catastrophe frequency of detached microtubules was 0.022 events per sec in the wild type, whereas it was decreased to 0.010 events per sec in the *nek6-1* mutant (Table S1). These results demonstrate that NEK6 is required to suppress distorted microtubules generated by detachment from the cortex. As dissociation frequency was not altered in *nek6-1*, NEK6 may not be essential for the anchoring of cortical microtubules to the plasma membrane.

It is possible that loss of function of NEK6 affects dynamic instability of individual microtubules, causing disorganized cortical microtubule arrays. Thus, we analyzed microtubule dynamics in the wild type and *nek6-1* constitutively expressing GFP-TUB6 (Table S2). The dynamics of the plus end of individual microtubules was analyzed in the epidermal cells of cotyledons, because microtubules are more sparse and clearly observed in cotyledons than hypocotyls, in which microtubules are more bundled. In addition, the ectopic outgrowths were also observed on the abaxial side of cotyledons. Thus, cotyledons are amenable to the analysis of individual microtubules. The parameters of microtubule dynamic instability were almost identical to those in the wild type (Table S2). Therefore, disorganization of cortical microtubules in the *nek6-1* mutant could not be attributed to the changes in overall microtubule dynamics. In summary, NEK6 may function as a specific regulator of a subpopulation of microtubules (e.g. eliminating aberrant microtubules generated by detachment events) rather than a general factor for dynamic instability of all microtubules.

### NEK6 overexpression disorganizes cortical microtubules and suppresses cell elongation

**Figure 4:**
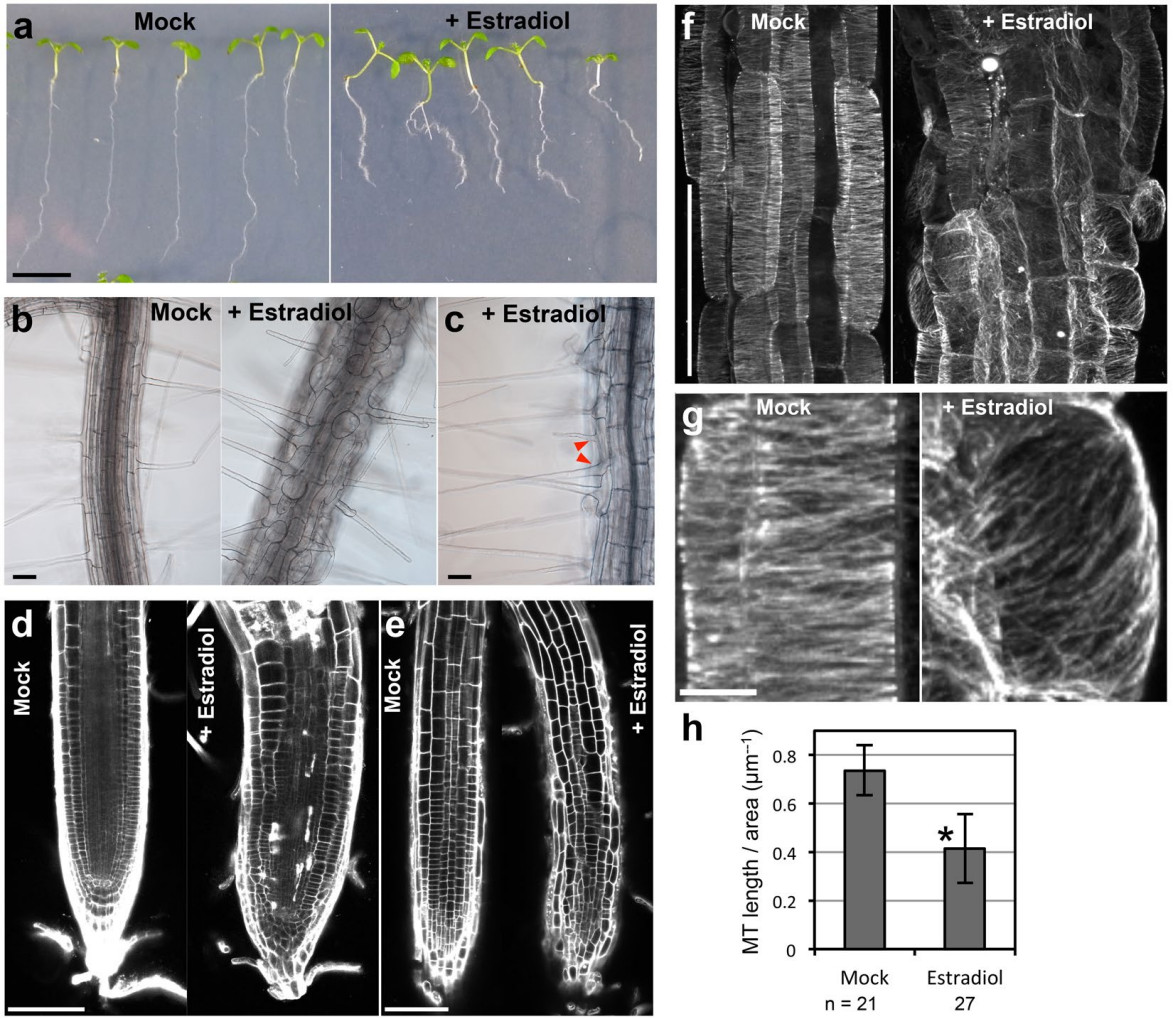
*NEK6* overexpression disorganizes cortical microtubules and suppresses cell elongation. (**a**) Morphology of seedlings harboring estrogen-inducible *NEK6*. Seedlings were grown vertically for 8 days in the absence (Mock) or presence of estradiol (+Estradiol). The scale bar represents 1 cm. (**b**,**c**) Effect of *NEK6* overexpression on epidermal cell morphology. Roots of seedlings grown for 8 days without (Mock) or with estradiol (+Estradiol) were observed under a microscope. Arrowheads indicate two root hairs in a trichoblast in the presence of estradiol. Scale bars represent 100 µm. (**d**,**e**) Middle-vertical section (**d**) and epidermal cells (**e**) of the root tip. The roots of seedlings grown for 8 days without (Mock) or with estradiol (+Estradiol) were stained with propidium iodide and observed under a confocal microscope. Scale bars represent 100 µm. (**f**) Effect of *NEK6* overexpression on cortical microtubule arrays. The seedlings grown for 4 days without (Mock) or with estradiol (+Estradiol) were stained with anti-β-tubulin antibody. Fluorescence images show cortical microtubules in root epidermal cells in the elongation region. The scale bar represents 100 µm. (**g**) Enlarged view of cortical microtubule arrays in (**f**). The scale bar represents 10 µm. (**h**) Quantification of cortical microtubule density without (Mock) or with estradiol (+Estradiol). Data are displayed as averages ± SDs. The asterisk indicates significant difference from the value in mock-treated plants (*t*-test, *P* < 0.001).

Next, we analyzed gain-of-function phenotypes caused by *NEK6* overexpression. Since we were unable to obtain stable transgenic lines constitutively overexpressing *NEK6* under the control of the *Cauliflower Mosaic Virus 35S* (*CaMV35S*) promoter, we used an estradiol induction system (an estrogen receptor-based XVE transactivator system)^29^. In transgenic plants harboring estrogen-inducible *NEK6*, estradiol treatment induced the expression of NEK6 approximately 100- to 1000-fold (Fig. S2a). When *NEK6* was induced by estradiol, root growth was severely inhibited (Fig. 4a). In addition, the seedlings showed a right-slanting root growth phenotype in the presence of estradiol (Fig. 4a). We also found that the effects of *NEK6* overexpression were almost completely reversible. The root growth was recovered by removal of estradiol, whereas root growth was affected when the seedlings were transferred to medium containing estradiol (Fig. S2b).

By contrast, shoot morphology of estradiol-treated transgenic plants was relatively normal compared with that of mock-treated plants (Fig. 4a). This might be due to insufficient incorporation of estradiol into shoot tissues and/or degradation of estradiol during root-to-shoot transport. In addition, it is also possible that microtubule-stabilization mechanism may counteract the effect of NEK6 induction in shoots and/or tubulin phosphorylation by NEK6 may be saturated in aerial organs (see below).

We subsequently examined the cell morphology and organization of cortical microtubules in estradiol-treated seedlings. Epidermal and cortical cells in the root elongation zone were radially swollen (Fig. 4b–e) and, occasionally, two root hairs developed from a single trichoblast cell (Fig. 4c). Transgenic seedlings overexpressing *NEK6* showed a disorganized root apical meristem after induction with estradiol, and contained cells that had an aberrant morphology and an irregular arrangement (Fig. 4d,e). Next, we analyzed microtubule organization through tubulin immunolabeling. The root epidermal cells of seedlings treated with estradiol had fewer and more disorganized cortical microtubules than non-treated cells (Fig. 4f–h). The density of cortical microtubules was significantly reduced in estradiol-treated seedlings (Fig. 4i). These results demonstrate that *NEK6* overexpression disorganizes cortical microtubules and severely suppresses cell elongation in roots.

### NEK6 localizes to the ends of shrinking microtubules

We previously observed that functional NEK6-GFP localizes to particles that associate with cortical microtubules and exhibit dynamic behaviors^24^. However, it was unclear how NEK6 moves on microtubules. To address this issue, we generated a transgenic *Arabidopsis* line that expresses both NEK6-GFP and mCherry-TUB6, and monitored these markers through live cell imaging.

**Figure 5:**
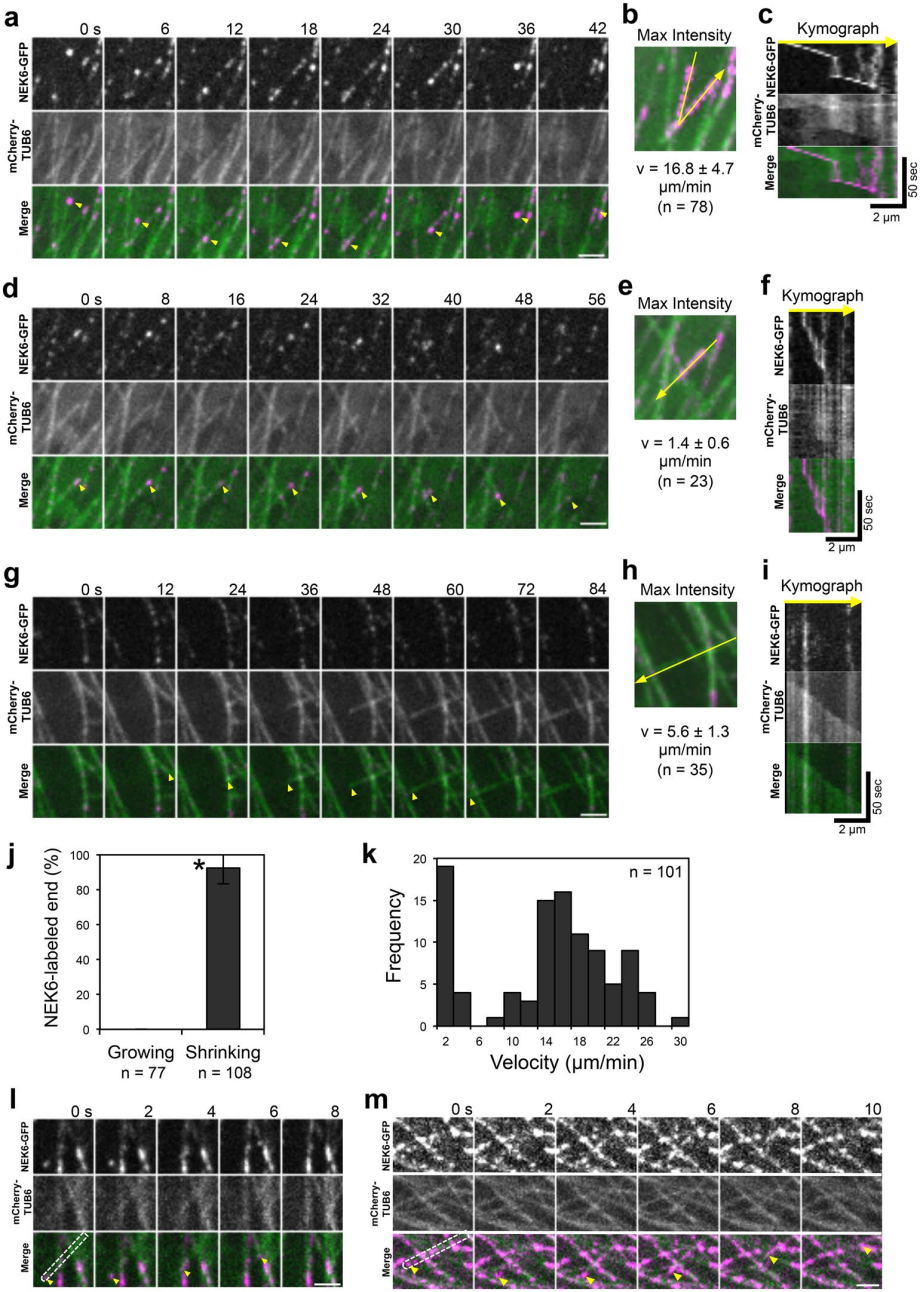
NEK6 Localizes to the shrinking microtubule ends. (**a**) A time-lapse montage showing that NEK6-GFP localizes to shrinking microtubule plus end. Arrowheads indicate the shrinking plus end of a microtubule. The scale bar represents 2 µm. (**b**) A maximum intensity projection of (**a**). The yellow line shows the location of the kymograph line. (**c**) Kymograph of the line in (**b**). (**d**) A time-lapse montage showing that NEK6-GFP localizes to the shrinking microtubule minus end. Arrowheads indicate the shrinking minus end of a microtubule. The scale bar represents 2 µm. (**e**) A maximum intensity projection of (**d**). The yellow line shows the location of the kymograph line. (**f**) Kymograph of the line in (**e**). (**g**) A time-lapse montage showing that NEK6-GFP does not localize to the growing plus end. Arrowheads indicate the growing plus end of a microtubule. The scale bar represents 2 µm. (**h**) A maximum intensity projection of (G). The yellow line shows the location of the kymograph line. (**i**) Kymograph of the line in (H). (**j**) The percentage of growing or shrinking ends of microtubules labeled with NEK6-GFP. Data are displayed as averages ± SD. The asterisk indicates significant difference from the value in the growing ends (*t*-test, *P* < 0.001). (**k**) Histogram of shrinking rates for NEK6-GFP-labelled microtubules. (**l**, **m**) A time-lapse montage showing that NEK6-GFP localizes to the shrinking end of a detached and distorted cortical microtubule. Arrowheads indicate the NEK6-GFP-localized shrinking end. The areas enclosed by broken lines indicate the detached microtubules. The scale bar represents 2 µm. RFP signals are shown in green and GFP signals are shown in magenta in (**a**–**i**,**l**,**m**).

NEK6 particles were frequently observed at the microtubule ends and intersections. NEK6-GFP-labeled microtubule ends rapidly disassembled and did not grow (Fig. 5a–j and Movies S6 and S7). Most of the growing ends of microtubules were not labeled with NEK6-GFP (Fig. 5g–j and Movie S7). NEK6-GFP associated with both rapid- and slow-shrinking ends (Fig. 5a–f,k and Movie S6). Thus, NEK6 preferentially localized to the shrinking ends of microtubules. In addition, NEK6-GFP was frequently transferred from one shrinking microtubule to another (Fig. 5a–c and Movie S6). Thus, NEK6 particles may switch microtubule tracks. As an exceptional case, NEK6-GFP particles localized to both the growing and shrinking microtubule ends in the basal region of hypocotyls (near the root-hypocotyl junction, Fig. S3 and Movie S8). Cortical microtubules were sparse and longitudinally oriented in this region. These NEK6-GFP-labeled ends tended to grow towards the edge of the cell.

The increase of detached microtubules in the *nek6* mutant prompted us to examine whether NEK6-GFP localizes to the shrinking ends of microtubules detached from the cell cortex. Although the number of detached microtubules was limited in the wild type and NEK6-GFP complementation lines, we carefully monitored NEK6-GFP and mCherry-TUB6 and found that NEK6-GFP localized to the shrinking ends of detached microtubules (Fig. 5l,m and Movies S9 and S10). This result supports our hypothesis that NEK6 suppresses distorted cortical microtubules generated by detachment from the cortex.

### Phosphorylation sites of β-tubulin by NEK6 *in vitro*

**Figure 6:**
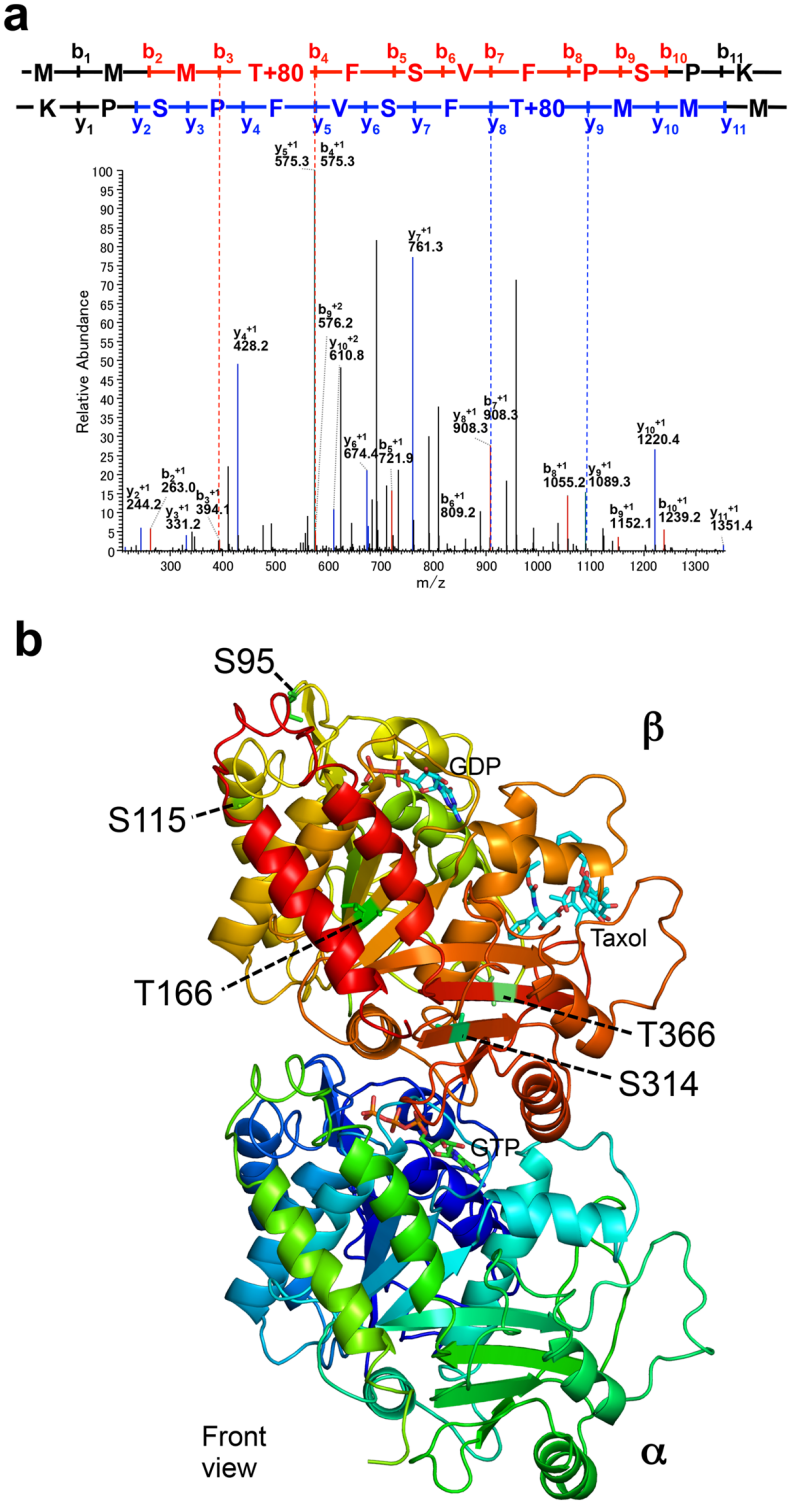
Phosphorylation sites of β-tubulin. (**a**) MS/MS spectrum of a phosphopeptide (163-MMMpTFSVFPSPK-174) from NEK6-treated TUB4. (**b**) NEK6 phosphorylation sites of β-tubulin. Five phosphorylation sites are shown on the structure of α/β-tubulin heterodimer. The tubulin structure is shown as a ribbon diagram (front view from the outside of the microtubule).

We previously showed that NEK6 phosphorylates native tubulin and recombinant β-tubulin *in vitro* ^24^. However, the function and sites of phosphorylation remained to be elucidated. Therefore, we identified the amino acid residues of β-tubulin that are phosphorylated by NEK6. We expressed *Arabidopsis* β-tubulin 4 (TUB4) and α-tubulin 4 (TUA4) proteins tagged with 6xHis in *Escherichia coli* and purified the tagged proteins by affinity chromatography. These proteins were used in an *in vitro* kinase assay with recombinant glutathione S-transferase (GST)-NEK6 protein (Fig. S4a). GST-NEK6 strongly phosphorylated TUB4 but not TUA4, indicating that NEK6 specifically phosphorylates β-tubulin. To determine the specific phosphorylation sites of β-tubulin, recombinant TUB4 was phosphorylated by GST-NEK6 *in vitro*, digested with trypsin, and subjected to LC-MS/MS analysis. We detected phosphorylated peptides derived from TUB4 in the LC-MS/MS spectra and determined five phosphorylation sites in TUB4 (Figs 6a and S4b–e). These phosphorylation sites were mapped on the 3D-structure of the α/β-tubulin heterodimer^30^ (Fig. 6b). Ser95 is involved in longitudinal interaction between tubulin dimers (i.e., the interdimer interaction site). Ser115 localizes to the lateral interface between microtubule protofilaments. Thr166 localizes to the β-sheet near the GTP/GDP binding site. Ser314 and Thr366 are near the interface between α-tubulin and β-tubulin (i.e., the intradimer interaction site). These phosphorylation sites are conserved in the β-tubulin family, but not in the α-tubulin family of plants and other organisms. We noticed that Ser115 is partially conserved in the *Arabidopsis* β-tubulin family (three β-tubulin members, TUB1, TUB5, and TUB6, do not have this phosphorylation site, whereas the remainder do). In the animal β-tubulin family, four phosphorylation sites are well-conserved (i.e., Ser95, Ser115, Thr166, and Thr366). Ser314 is conserved in plants and filamentous fungi (*Aspergillus* and *Neurospora*), but substituted with Ala in animals and yeast. Phosphorylation of these four sites has been detected by phosphoproteomic analysis of HeLa cells^31^. Phosphorylation of Thr166 has been reported in the plant phosphoproteomic database^32^. *Arabidopsis* β-tubulin mutants of Ser95 exhibit right-handed helical growth and this phenotype is recapitulated by ectopic expression of mutant β-tubulin proteins, which were incorporated into microtubules^33^. Mutations in the residues 165 and 167 (adjacent to T166) of fungus β-tubulin alter the sensitivity to microtubule depolymerizing benzimidazoles, suggesting involvement of this region in benzimidazole binding and microtubule stability^34–36^.

### Involvement of Thr166 in cortical microtubule depolymerization

**Figure 7:**
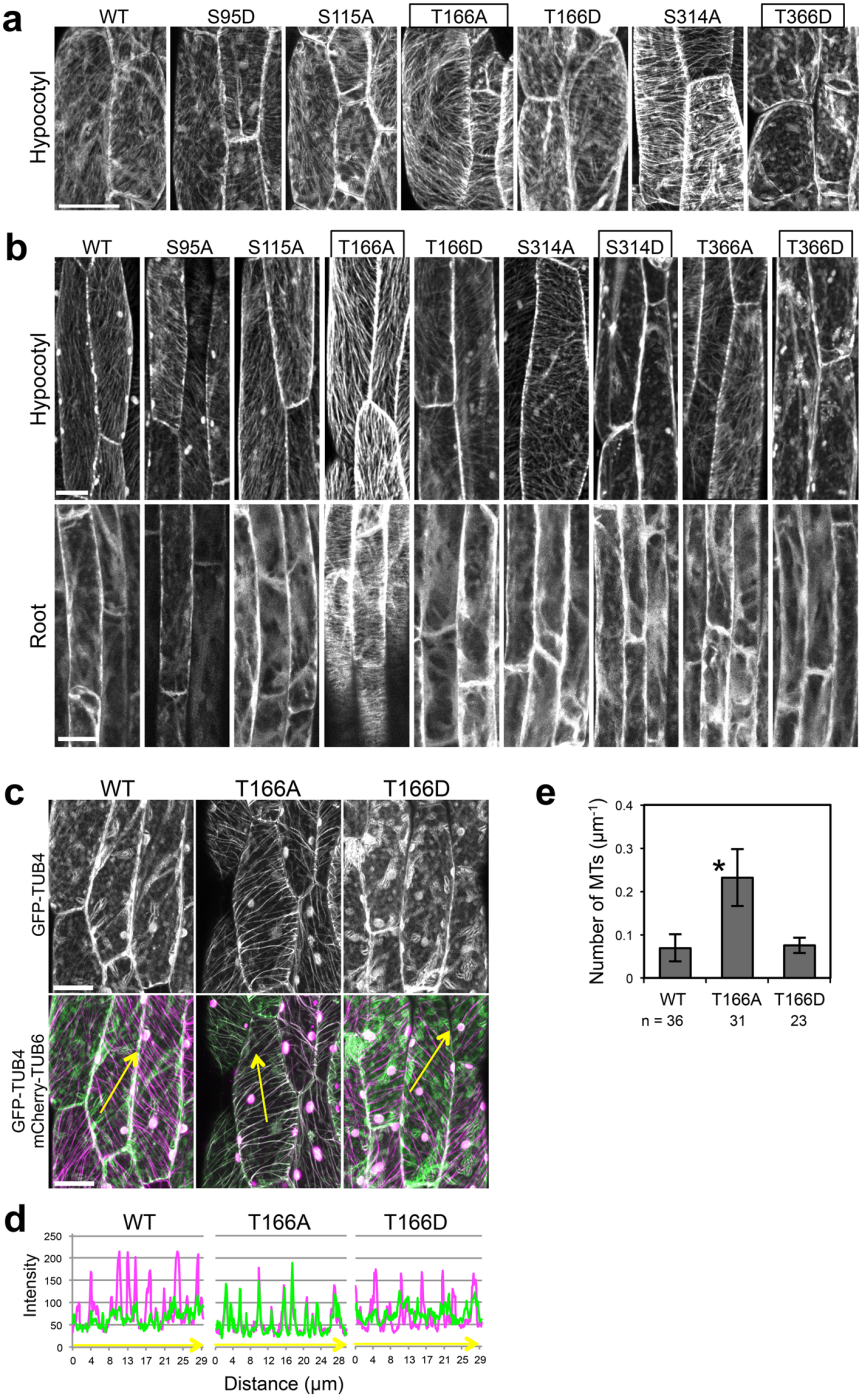
Involvement of Thr166 of β-tubulin in cortical microtubule depolymerization. (**a**,**b**) Subcellular localization of wild type TUB4 (WT) and mutated TUB4 proteins (S95A, S115A, T166A, T166D, S314A, S314D, T366A and T366D) fused with GFP at the N-terminus. GFP-TUB4 was constitutively driven by the CaMV35S promoter in (**a**) (*35S:GFP-TUB4*) or expressed under the *TUB4* own promoter in (**b**) (*TUB4pro:GFP-TUB4*). The scale bars represent 30 µm in (**a**) or 20 µm in (**b**). (**c**) Subcellular localization of wild type TUB4 (WT) and mutated TUB4 proteins (T166A or T166D) fused with GFP at the N-terminus (green). Cortical microtubules are visualized with mCherry-TUB6 (magenta). The scale bars represent 20 µm. (**d**) Profiles of fluorescence intensity of GFP and mCherry along lines shown in (**c**). (**e**) Density of microtubules labeled with the wild type TUB4 (WT) or mutated TUB4 (T166A or T166D) fused with GFP. Data are displayed as averages ± SD. The asterisk indicates significant difference from the value in wild type GFP-TUB4 (*t*-test, *P* < 0.001).

The phosphorylation sites of β-tubulin might be involved in the NEK6-mediated microtubule depolymerization. Therefore, we replaced the phosphorylation sites of TUB4 with a non-phosphorylatable alanine or a phosphomimetic aspartate. We then fused these mutated TUB4 proteins with GFP and expressed them under the *CaMV35S* promoter to monitor their localization. We successfully generated stable transgenic lines for six of the ten different substitutions (i.e., S95D, S115A, T166A, T166D, S314A, and T366D), but could not recover stable transgenic lines expressing GFP-TUB4 with the S95A, S115D, S314D or T366A substitution. Among the six substitution lines, two exhibited striking differences in GFP-TUB4 localization. The alanine substitution of Thr166 strongly promoted localization of the mutant TUB4 on cortical microtubules, whereas the aspartate substitution of Thr166 did not affect it (Fig. 7a). The aspartate substitution of Thr366 inhibited incorporation of the mutant tubulin into cortical microtubules and resulted in cytoplasmic localization of GFP-TUB4 (Fig. 7a). The other three substitutions (S95D, S115A, and S314A) did not strongly affect the localization of GFP-TUB4 (Fig. 7a).

In the course of this experiment, it is noticed that constitutive expression of GFP-TUB4 under the *CaMV35S* promoter suppressed seedling growth and survival, which is crucial for the efficient recovery of stable transgenic plants. Thus, we generated transgenic lines expressing GFP-TUB4 under the control of *TUB4* own promoter and analyzed their subcellular localization (Fig. 7b). We successfully generated stable transgenic lines for eight of the ten different substitutions (i.e., S95A, S115A, T166A, T166D, S314A, S314D, T366A, and T366D). Because we could not recover multiple stable transgenic lines expressing GFP-TUB4 with the S95D or S115D substitution, these mutant versions were excluded from the analysis. Wild-type GFP-TUB4 incorporated into cortical microtubules in the cells of aerial parts but not in the root cells (localized in the cytosol), suggesting the suppression of incorporation of GFP-TUB4 in roots (Fig. 7b). Localization patterns of mutated GFP-TUB4 were most identical with that of *CaMV35S*-driven GFP-TUB4. T166A remarkably promoted cortical microtubule localization of GFP-TUB4, especially in root cells, whereas T166D did not affect it (Figs 7b and S5). T366D inhibited microtubule localization of GFP-TUB4, resulting in the cytoplasmic localization (Fig. 7b). As a new finding, S314D also inhibited microtubule localization of GFP-TUB4, same in the case of T366D (Fig. 7b), implying that phosphorylation of S314 and T366 may promote microtubule depolymerization. It is noteworthy that TUB4pro:GFP-TUB4^T166A^ can be a useful tool for analyzing microtubule organization in root cells, because GFP-TUB4^T166A^ strongly labeled microtubules in root cells but not affected plant growth and morphology (Movies S11 and 12). Previous GFP-tubulin reporters did not well incorporated into cortical microtubules of root cells, and some microtubule markers cause helical growth of organs^37, 38^. In summary, these results identify Thr166 as a major regulatory site, which strongly affects microtubule localization of TUB4.

We subsequently co-expressed GFP-TUB4 and mCherry-TUB6 to confirm that T166A substitution promotes the microtubule localization of GFP-TUB4 (Fig. 7c,d). Wild-type GFP-TUB4 colocalized with mCherry-TUB6 to a lesser extent than did T166A-substituted GFP-TUB4, whereas T166A-substituted GFP-TUB4 was strongly colocalized with mCherry-TUB6 in the cortical microtubules. The T166D substitution did not affect the localization of GFP-TUB4 (Fig. 7c,d). This result was confirmed by the quantification of cortical microtubule density (Fig. 7e). Microtubule localization was increased in T166A-substituted GFP-TUB4 by about 3-fold compared with those in the wild-type GFP-TUB4 and T166D-substituted GFP-TUB4.

It is noteworthy that wild-type GFP-TUB4 was predominantly localized to the cytoplasm, whereas mCherry-TUB6 and GFP-TUB6 was not, suggesting that TUB4 might be susceptible to phosphorylation and depolymerization by NEK6. To examine whether phosphorylatable TUB4 could be remained on the cortical microtubules, we compared the ratio of fluorescence signal intensity of cytoplasmic free GFP-TUB4 to that of microtubule-localized GFP-TUB4 between wild type and T166D-substituted GFP-TUB4 (Fig. S6). The ratio of fluorescence intensity of cytoplasmic free tubulin to that of microtubules was slightly but not significantly increased in T166D-substituted GFP-TUB4. This result suggests that most of the wild type GFP-TUB4 is phosphorylated at Thr166 and then depolymerized (i. e. saturation of TUB4 phosphorylation by NEK6), but we cannot exclude small amount of phosphorylatable TUB4 on the cortical microtubules. In addition, there are some technical problems such that it is difficult to detect separately the fluorescence of cytoplasmic free tubulin and microtubules in the whole cell and to determine precise ratio of cytoplasmic free tubulin to microtubule-associated tubulin.

### NEK6 kinase activity is essential for directional cell growth

**Figure 8:**
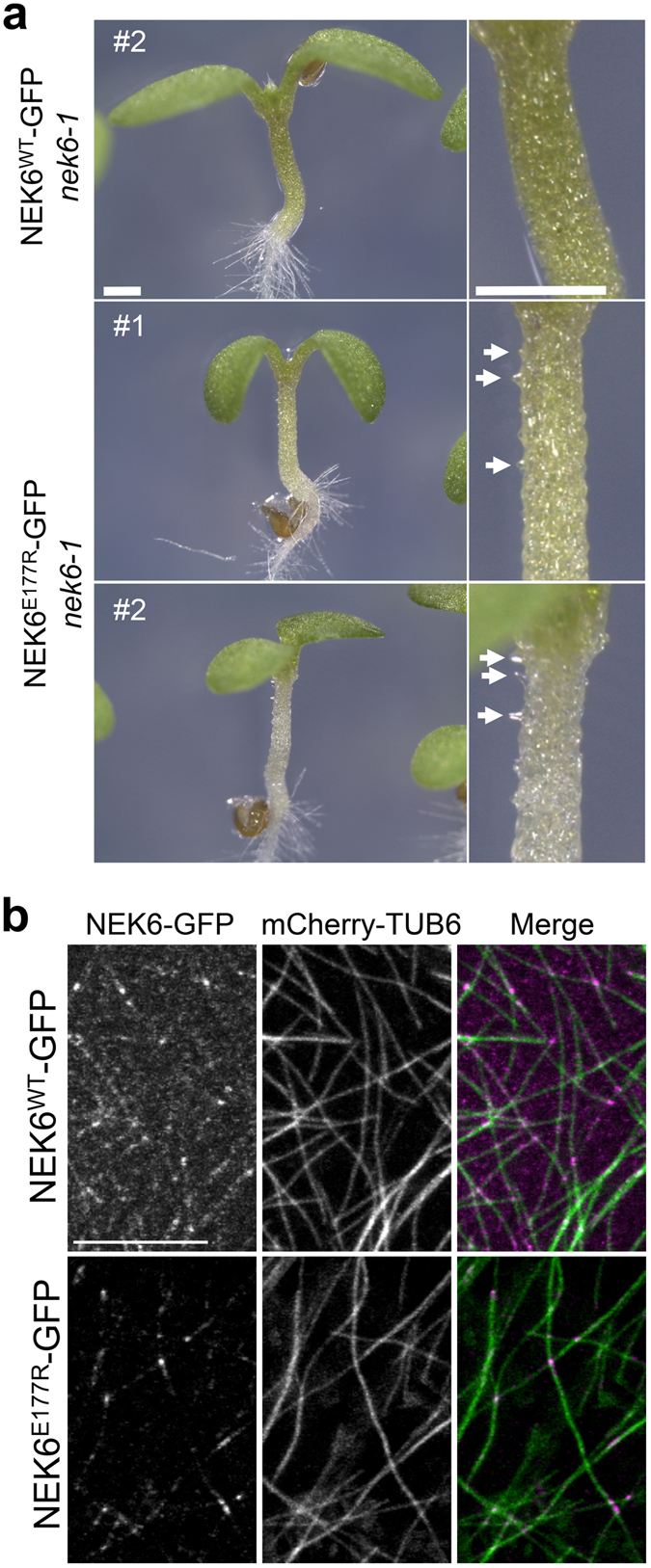
NEK6 kinase activity is essential for directional cell growth. (**a**) The ectopic outgrowth phenotype is complemented by expressing the wild type NEK6 (WT) but not by expressing the kinase-dead NEK6 (E177R). The scale bars represent 0.5 mm. (**b**) Subcellular localization of wild type NEK6 (WT) and kinase-dead NEK6 (E177R) proteins fused with GFP at the C-terminus (magenta). Cortical microtubules are visualized with mCherry-TUB6 (green). RFP signals are shown in green and GFP signals are shown in magenta. The scale bar represents 10 µm.

Next, we analyzed whether the kinase activity of NEK6 is indispensable for its function in directional cell expansion. We previously identified a kinase-dead allele of NEK6 named *ibo1-1*, which has a substitution of Arg for Glu177 (E177R) at the C-terminal region of the activation loop^22^. The *ibo1-1* mutation (E177R) was introduced into NEK6-GFP, and then both of the wild type and mutant NEK6-GFP fusion proteins were expressed in the *nek6-1* mutant under the control of *NEK6* promoter. The wild type NEK6-GFP (NEK6^WT^-GFP) complemented the ectopic outgrowth phenotype of *nek6-1* (Fig. 8a, n = 64/64, five independent lines), whereas the kinase-dead mutant NEK6^E177R^-GFP did not complement the *nek6-1* phenotype (Fig. 8a, n = 90/90, six independent lines). This result demonstrates that NEK6 kinase activity is essential for directional cell expansion.

To determine whether the kinase activity is required for microtubule localization of NEK6, we analyzed subcellular localization of NEK6^WT^-GFP and NEK6^E177R^-GFP. Cortical microtubules were simultaneously observed with mCherry-TUB6. Both of NEK6^WT^-GFP and NEK6^E177R^-GFP localized to the microtubule lattice and microtubule crossover sites (Fig. 8b). Thus, NEK6 kinase activity is not essential for its microtubule localization. This is consistent with our previous result that NEK6 C-terminal region is mainly required for its microtubule localization^22^. Interestingly, we noticed that NEK6^E177R^-GFP signal was remarkably weaker compared with that of NEK6^WT^-GFP (Fig. 8b), implying that the kinase activity may be required to stabilize NEK6 protein probably through autophosphorylation. Because of the weak signal of NEK6^E177R^-GFP, we could not detect localization of NEK6^E177R^-GFP to the shrinking ends of microtubules.

## Discussion

In this study, we showed that *Arabidopsis* NEK6 maintains polar plant growth by promoting longitudinal cell expansion and suppressing ectopic outgrowth. Both of these functions may be mediated by NEK6-dependent microtubule disassembly. The *nek6-1* mutant exhibits a disorganized cortical microtubule array, in which microtubule bending and bundling are frequently observed. These abnormal microtubules can be removed directly or indirectly by NEK6. It is possible that NEK6 directly recognizes aberrant microtubules and specifically depolymerizes them. Several end-tracking MAPs associate with the shrinking ends of microtubules to promote local microtubule disassembly during secondary cell wall patterning^39^ and in yeast chromosome segregation^40^. Loss of NEK6 function results in the increase of microtubule distortion concomitant with detachment of microtubules from the cortex, supporting this hypothesis. The partial detachment of microtubules promotes searching capacity of microtubules, which results in the increase of microtubule interactions and bundling manifested in the *clasp* mutant^28^. Thus, CLASP activity regulates microtubule reorganization through promoting microtubule-cortex attachment. Because microtubule detachment frequency in the *nek6* mutant is same as in the wild type, NEK6 may promote depolymerization of aberrant microtubules after detachment from cortex. It seems contradictory that the *clasp* mutant has longer detached microtubules^28^ but does not show any ectopic outgrowth in epidermal cells^41, 42^. Because the *clasp* mutant exhibits a severe dwarf phenotype mainly due to suppression of cell division and elongation^41, 42^, ectopic outgrowth may also be suppressed in *clasp* mutant. In addition, the *clasp* mutant shows suppression of lobe outgrowth in epidermal pavement cells^41, 42^ but relatively normal microtubule organization in hypocotyl epidermal cells^42^. These phenotypes are different from those observed in *nek6* mutants, suggesting distinct developmental roles of CLASP and NEK6. In summary, NEK6 may participate in microtubule surveillance system to reduce excessive distortion of microtubules after detachment from the cortex.

Another possibility, which does not exclude the first one, is that NEK6-dependent depolymerization reduces microtubule density, thereby maintaining the optimal number of microtubules in the cell. In the absence of this regulatory mechanism, cortical microtubules are stabilized, microtubule density is increased, and microtubule-microtubule interactions are promoted, which may lead to aberrant cortical microtubule arrays. The spatial density of microtubules is a critical parameter that affects the self-organizing property of cortical microtubules based on encounter-angle-dependent microtubule bundling or catastrophe^4, 43^. It would be interesting to determine whether NEK6 destabilizes specific microtubules and/or affects microtubule self-organizing behaviors. Taken together, our results demonstrate that NEK6 coordinates directional cell growth predominantly through depolymerization of cortical microtubules (Fig. S7).

We identified five β-tubulin sites that are phosphorylated by NEK6 *in vitro*. Although these phosphorylation sites may function cooperatively in microtubule destabilization, Thr166 is likely to be a major phosphorylation target of NEK6, because the non-phosphorylatable T166A mutation strongly enhanced tubulin incorporation into microtubules (Fig. 8) and phosphorylation of Thr166 has been detected in phosphoproteomic analyses *in vivo* ^31, 32^. NEK6 directly binds to microtubules *in vitro* and localizes to cortical microtubules *in vivo* ^24^, implying that NEK6 phosphorylates polymerized tubulin to destabilize existing microtubules. However, we could not exclude a possibility that NEK6 phosphorylates cytoplasmic free tubulin to regulate the level of active tubulin heterodimers as in the case of *Arabidopsis* tubulin kinase PHS1^13–15^. In our analysis, about half of GFP-TUB4 localized in the cytoplasm and microtubule fluorescence was almost the same between the wild-type GFP-TUB4 and phosphomimic GFP-TUB4^T166D^, suggesting few amount of phosphorylatable TUB4 on microtubules and/or saturation of TUB4 phosphorylation by NEK6. Because the phosphorylation sites are well conserved in the β-tubulin family and NEK6 also phosphorylates TUB6 *in vitro* ^24^, different β-tubulin isotypes could be the target of microtubule-localized NEK6.

PHS1 phosphorylates Thr349 of α-tubulin of unpolymerized tubulin dimers in response to osmotic stress and suppresses the assembly of tubulin dimers into microtubules^13–15^. Thr349 of α-tubulin is located at the interdimer interaction site and its phosphorylation is expected to interfere with the longitudinal interaction between two adjacent tubulin dimers^13, 14^. On the other hand, Thr166 is located at the middle region of the fifth β sheet (B5) near the ribose of GDP^30^. This region, which includes B5, contributes to the binding of GTP to β-tubulin. Photoaffinity labeling of tubulin with [α-^32^P]GTP indicates that the β-tubulin sequence region (residues 155–174) forms a GTP-binding site^44^. In addition, an antibody to the peptide of residues 154–165 inhibits GTP incorporation^44^. Therefore, phosphorylation of Thr166 may affect the GTP/GDP binding affinity and GTP/GDP exchange of β-tubulin. A similar mechanism has been proposed for the phosphorylation of β-tubulin by cyclin-dependent kinase (Cdk) during the M-phase in animal cells. Cdk phosphorylates Thr172 of unpolymerized tubulin and impairs its assembly into microtubules^45^. Thus, NEK6 provides a novel mechanism for microtubule depolymerization based on the phosphorylation of tubulin. Thr166 and other phosphorylation sites are well conserved within the β-tubulin family of plants, fungi, and animals, and several phosphorylation sites have been detected by phosphoproteomic analyses^31, 32^. Considering that *Aspergillus* NIMA regulates microtubule organization in directional cell growth during interphase^21^ and human NEK6 and NEK7 phosphorylate microtubule preparation *in vitro* ^46^, this mechanism might be conserved in other organisms, including fungi and animals.

Recent studies revealed that microtubules are regulated at specific sites in the cortical microtubule array (plus/minus ends, branching sites, and crossover sites). Several microtubule-associated proteins (MAPs) recognize these sites and locally regulate the dynamics and organization of microtubules^5–11^. NEK6 also specifically recognizes microtubule ends, at which multiple phosphorylation sites are exposed on the surface of β-tubulin. Our results imply that posttranslational modification of microtubules at the specific sites (microtubule ends in this case) alters microtubule organization and behavior. This notion is consistent with the tubulin code hypothesis, in which posttranslational modifications of tubulin (phosphorylation, detyrosination/tyrosination, polyglutamylation, polyglycylation, and acetylation) are proposed to chemically mark distinct microtubule subpopulations and adjust their structures and behaviors for specific functions^47^. However, our finding does not exclude the possibility that NEK6 functionally interacts with and phosphorylates other MAPs to depolymerize cortical microtubules. Indeed, it has been shown that the plant-specific Armadillo-Repeat Kinesin 1 (ARK1) interacts with NEK6^23^ and localizes to the growing microtubule plus ends to promote microtubule catastrophe^48^. ARK1 and other MAPs may cooperate with NEK6 to modulate microtubule stability. In conclusion, we propose a novel regulatory mechanism of acentrosomal cortical microtubule arrays in which NEK6 phosphorylates β-tubulin to promote microtubule depolymerization. The NEK6-induced depolymerization coordinates cortical microtubule organization and directional cell growth.

## Methods

### Plant material and growth conditions

*Arabidopsis thaliana* accession Colombia, *nek6-1* (*ibo1-4*)^22^, NEK6-GFP^24^, GFP-TUB6^49^, and mCherry-TUB6^7^ were used in this study. Surface-sterilized seeds were sown on half-strength Murashige and Skoog (MS) agar medium and vertically incubated under a 16 h-light/8 h-dark photoperiod at 23 °C^50^. In estradiol treatment, seeds were germinated on MS medium supplemented with or without 10 µM estradiol. During seedling-transfer experiments, seedlings grown for 7 days on the MS medium with or without 10 µM estradiol were transferred onto the MS medium with or without 10 µM estradiol.

### Vectors and transgenic plants

Vectors were constructed based on Gateway technology (Life Technology). For NEK6 induction, the full-length coding sequence of NEK6 was amplified by PCR and cloned into the pENTR/D-TOPO cloning vector^24^. The entry clone was recombined with pER8 containing a Gateway cassette (pMDC7), which was generated as described previously^51^.

The NEK6 genomic fragment (2104-bp promoter region and coding region without the stop codon) was previously subcloned into pENTR-D-TOPO^24^. Site directed mutagenesis was conducted to substitute Glu177 with arginine by using the KOD mutagenesis kit (TOYOBO, http://lifescience.toyobo.co.jp). Primers used in the mutagenesis are listed in Table S3. The wild type and E177R mutant version were transferred to the Gateway binary vector pGWB504^52^ by the Gateway LR reaction (Invitrogen) to generate NEK6pro:NEK6^WT^-GFP and NEK6pro:NEK6^E177R^-GFP.

The cDNA encoding the full-length TUB4 or TUA4 cloned into pENTR223 was obtained from ABRC and transferred to pDEST17 by LR reaction to generate 6xHis-tagged TUB4 and TUA4 construct. Site directed mutagenesis was performed to substitute each phosphorylation site of TUB4 with alanine or aspartate by KOD mutagenesis kit (TOYOBO). Primers used in the mutagenesis are listed in Table S3. The wild-type and mutated TUB4 cDNA in pENTR223 were transferred to pGWB6^53^ by LR reaction to generate *CaMV35Spro:GFP-TUB4* constructs. The TUB4 promoter region (1535-bp region upstream of the initiation codon) was amplified from the wild type Columbia genomic DNA by PCR using the primers shown in Table S3 and cloned into the HindIII-XbaI site of pGWB6 to generate pGWB6-TUB4pro. The wild-type and mutated TUB4 cDNA in pENTR223 described above were transferred to pGWB6-TUB4pro by LR reaction to generate *TUB4pro:GFP-TUB4* constructs.

*Agrobacterium tumefaciens* strain GV3101 (pMP90) was used to transform *Arabidopsis* plants by floral dip method^54^. The pMDC7-NEK6 construct was transformed into the *Arabidopsis* wild type (Colombia accession). The NEK6pro:NEK6^WT^-GFP and NEK6pro:NEK6^E177R^-GFP constructs were introduced into the *nek6-1* mutant expressing mCherry-TUB6, which was introduced by crossing with *nek6-1*. The *CaMV35Spro:GFP-TUB4* and *TUB4pro:GFP-TUB4* constructs were introduced into the wild type (Colombia accession). To confirm localization pattern of GFP-TUB4, at least 10 independent transgenic lines were analyzed for each construct and similar results were obtained. To obtain seedlings expressing both GFP-TUB4 and mCherry-TUB6, *35Spro:GFP-TUB4* lines were crossed to the mCherry-TUB6 lines.

### RT-qPCR

Total RNA was isolated from whole seedlings grown for 8 days in the presence or absence of estradiol by phenol/chloroform extraction and subsequent lithium chloride precipitation^55^. For each sample, 0.5 µg of total RNA was reverse transcribed to cDNA using ReverTra Ace reverse transcriptase (TOYOBO, http://www.toyobo.co.jp) according to the accompanying protocol. Real-time PCR was performed on a thermal cycler Dice Real Time System (Takara) using KAPA SYBR FAST qPCR Kit (KAPA Biosystems, https://www.kapabiosystems.com) according to the manufacturer’s method. Transcript levels of *ACT8* were used as a reference for normalization. Primers used in RT-qPCR are listed in Table S3. The RT-qPCR were performed using at least three biological replicates.

### Microscopy

For quantification of cell length and ectopic outgrowth, the wild type and *nek6-1* mutant seedlings were grown on MS medium, stained with propidium iodide solution (50 µg ml^−1^) for 1 minute, washed with water three times, and observed under a confocal laser-scanning microscope (FV1200, Olympus, http://www.olympus-lifescience.com/ja/). Maximum projection of z-series images of hypocotyls were taken and analyzed by image processing software (ImageJ 1.49, http://imagej.nih.gov/ij/). Longitudinal cell length was measured in each epidermal cell in the z-stack images. To determine the number and length of ectopic protrusions, 3D images were reconstructed by an ImageJ plugin VolumeViewer (http://rsb.info.gov/ij/plugins/volume-viewer.html) and longitudinal optical sections were analyzed. The length of ectopic protrusions was measured in the optical sections of 3D images. The data were obtained from 10 cell files of 5 seedlings for each time point and genotype.

Microtubule dynamics was analyzed in cotyledon epidermal cells of 4-d old light grown seedlings expressing GFP-TUB6. Epidermal cells were observed under a fluorescence microscope (ECLIPSE Ti, Nikon, http://www.nikoninstruments.com) equipped with a spinning-disk confocal unit (CSU-X, Yokogawa, http://www.yokogawa.co.jp/scanner/index.htm) and EM-CCD camera (DU897EM, Andor, http://www.andor.com). For time-lapse experiments, 1-sec exposures were acquired every 2 or 3 sec during the course of 300 sec using approximately 3.5 mW of excitation power, as measured at the end of the fiber optic delivering light to the scan head. Acquisition was controlled by MetaMorph (Molecular Devices, http://www.moleculardevices.com), and images were processed and analysed with ImageJ 1.49. Detached microtubules were detected by their lateral swinging movement and/or moving away from the focal plane.

For double labeling of NEK6 and microtubules, NEK6-GFP transgenic lines were crossed to the mCherry-TUB6 lines and seedlings expressing both reporters were analyzed. Epidermal cells of hypocotyls and cotyledons of 4-day-old seedling were observed under a fluorescence microscope (ECLIPSE Ti) equipped with a spinning-disk confocal unit (CSU-X, Yokogawa) and EM-CCD camera (DU897EM, Andor) as described above.

Seedlings expressing GFP-TUB4 and mCherry-TUB6 were observed under a FV1200 confocal laser-scanning microscope. Microtubule density was quantified by counting the number of cortical microtubules that crossed a line segment drawn across the cellular area^13, 33^.

Indirect immunofluorescence staining was performed as previously described^38^ to visualize microtubules in 4-day-old wild type and transgenic plants harboring estradiol-inducible *NEK6* construct. Mouse monoclonal anti-β-tubulin antibody KMX-1 (Merck Millipore, http://www.emdmillipore.com) was used as a primary antibody at the dilution of 1/100. Alexa Fluor 488-conjugated anti-mouse antibody was used as a secondary antibody at a dilution of 1/300. Root epidermal cells were observed under a FV1200 confocal laser-scanning microscope. Z-stack images were taken at 0.5 µm intervals.

### Purification of recombinant proteins and kinase assay

GST-NEK6, 6xHis-TUB4, and 6xHis-TUA4 proteins were expressed in *E*. *coli* and purified as previously described^24^. GST-NEK6 and 6xHis-TUB4/TUA4 constructs were previously described^22, 24^. Each construct was transformed into *Escherichia coli* strain KRX (Promega). Recombinant proteins were induced by the addition of 0.2 mM isopropyl 1-thio-β-galactopyranoside and 0.1% rhamnose and further incubation at 20 °C for 16 hr. *E*. *coli* cells expressing GST-NEK6 were recovered by centrifugation (6000 rpm for 5 min at 4 °C) and resuspended in a buffer (50 mM 2-amino-2-(hydroxymethyl)-1,3-propanediol (Tris)–HCl pH 8.0, 50 mM NaCl, 1 mM EDTA, 1 mM dithiothreitol). Cells expressing 6xHis-TUB4 or 6xHis-TUA4 were recovered by centrifugation and resuspended in a buffer (50 mM sodium phosphate buffer pH 7.0, 6 M guanidine-HCl, 300 mM NaCl). Cells were lysed using a French press (SLM Instruments, Inc). The lysate was centrifuged at 15000 rpm for 15 min at 4 °C and the supernatant was used for the affinity purification. GST-NEK6 were purified with Glutathione-Sepharose 4B (GE Healthcare) according to the manufacturers’ instruction. The His-tagged TUB4 or TUA4 were purified with TALON metal affinity resin (Clontech) according to the manufacturers’ instruction. The purified recombinant TUB4 or TUA4 were subjected to step-wise dialysis against 50 mM Tris-HCl pH 7.2, 1 mM dithiothreitol with 4 M, 2 M, and 1 M guanidine-HCl, and then subjected to three additional dialysis against 50 mM Tris-HCl pH 7.2 at 4 °C for 4 hr. One microgram of GST-NEK6 were incubated with or without 1 µg of His-TUB4 or His-TUA4 in 20 μl of kinase buffer [50 mM Tris-HCl pH 7.2, 15 mM MnCl_2_, 1 mM dithiothreitol, 10 μM ATP and 10 μCi [γ-^32^P]ATP] at 25 °C for 30 min. Reaction products were separated by SDS-PAGE followed by staining with Coomassie Brilliant Blue R250. Phosphorylated proteins were detected with an image analyzer (FLA-7000 IP, Fujifilm) and imaging plates (BAS-MS2040).

### LC-MS/MS

For LC-MS/MS analysis, 0.1 mg of His-tagged TUB4 were incubated with or without 0.1 mg GST-NEK6 in 100 µL kinase buffer [50 mM Tris–HCl pH 7.2, 15 mM MnCl_2_, 1 mM dithiothreitol, 1 µM ATP] at 25 °C for 30 min. To confirm phosphorylation of TUB4, a small aliquot (2 µl) from the kinase assay was separated by SDS-PAGE and stained with Pro-Q Diamond Phosphoprotein Gel Stain (Thermo Fisher, https://www.thermofisher.com/) to detect phosphorylated proteins followed by staining with Oriole Fluorescent Gel Stain (Bio-Rad, http://www.bio-rad.com/).

Phosphorylated samples were subjected to in-solution digestion with trypsin followed by LC-MS/MS analysis as previously described^56^. The reduction and alkylation steps were omitted. The sample solutions of kinase assay were digested by trypsin (12.5 ng µl^−1^, Sequencing Grade Modified Trypsin, Promega, https://www.promega.jp/) in the presence of 0.05% (w/v) ProteaseMAX^TM^ Surfactant (Promega) at 37 °C for 4 h. The digestion reaction was stopped by adding 0.5% (v/v) trifluoroacetic acid and incubating at 25 °C for 5 min. The resulting digests were purified by passing through a solid phase extraction tip (C-TIP, AMR, http://www.amr-inc.co.jp) and were injected to a Finningan LTQ liner ion trap mass spectrometer (Thermo). The data of MS and MS/MS were analyzed using BioWorks software version 3.3 (Thermo) with protein sequence information of *Arabidopsis* β-tubulins. The peptides detected by LC-MS/MS are listed in Table S4. The sequence coverage of 77% was achieved for TUB4.

## Electronic supplementary material

Supplementary Information

Movie S1

Movie S2

Movie S3

Movie S4

Movie S5

Movie S6

Movie S7

Movie S8

Movie S9

Movie S10

Movie S11

Movie S12

## References

[1] Mitchison T, Kirschner M (1984). Dynamic instability of microtubule growth. Nature.

[2] Kollman JM, Merdes A, Mourey L, Agard DA (2011). Microtubule nucleation by γ-tubulin complexes. Nat. Rev. Mol. Cell Biol..

[3] Hashimoto T (2015). Microtubules in Plants. Arabidopsis Book.

[4] Dixit R, Cyr R (2004). Encounters between dynamic cortical microtubules promote ordering of the cortical array through angle-dependent modifications of microtubule behavior. Plant Cell.

[5] Murata T (2005). Microtubule-dependent microtubule nucleation based on recruitment of γ-tubulin in higher plants. Nat. Cell Biol..

[6] Chan J, Sambade A, Calder G, Lloyd C (2009). *Arabidopsis* cortical microtubules are initiated along, as well as branching from, existing microtubules. Plant Cell.

[7] Nakamura M, Ehrhardt DW, Hashimoto T (2010). Microtubule and katanin-dependent dynamics of microtubule nucleation complexes in the acentrosomal *Arabidopsis* cortical array. Nat. Cell Biol..

[8] McNally FJ, Vale RD (1993). Identification of katanin, an ATPase that severs and disassembles stable microtubules. Cell.

[9] Lindeboom JJ (2013). A mechanism for reorientation of cortical microtubule arrays driven by microtubule severing. Science.

[10] Uyttewaal M (2012). Mechanical stress acts via katanin to amplify differences in growth rate between adjacent cells in *Arabidopsis*. Cell.

[11] Ambrose C, Allard JF, Cytrynbaum EN, Wasteneys GO (2011). A CLASP-modulated cell edge barrier mechanism drives cell-wide cortical microtubule organization in *Arabidopsis*. Nat. Commun..

[12] Naoi K, Hashimoto T (2004). A semidominant mutation in an *Arabidopsis* mitogen-activated protein kinase phosphatase-like gene compromises cortical microtubule organization. Plant Cell.

[13] Fujita S (2013). An atypical tubulin kinase mediates stress-induced microtubule depolymerization in *Arabidopsis*. Curr. Biol..

[14] Ban Y (2013). α-tubulin is rapidly phosphorylated in response to hyperosmotic stress in rice and *Arabidopsis*. Plant Cell Physiol..

[15] Hotta T (2016). Affinity purification and characterization of functional tubulin from cell suspension cultures of *Arabidopsis* and tobacco. Plant Physiol..

[16] Ben-Nissan G (2008). *Arabidopsis* casein kinase 1-like 6 contains a microtubule-binding domain and affects the organization of cortical microtubules. Plant Physiol..

[17] Fry AM, O’Regan L, Sabir SR, Bayliss R (2012). Cell cycle regulation by the NEK family of protein kinases. J. Cell Sci..

[18] Mahjoub MR (2002). The *FA2* gene of *Chlamydomonas* encodes a NIMA family kinase with roles in cell cycle progression and microtubule severing during deflagellation. J. Cell Sci..

[19] Bradley BA, Quarmby LM (2005). A NIMA-related kinase, Cnk2p, regulates both flagellar length and cell size in *Chlamydomonas*. J. Cell Sci..

[20] Osmani SA, Pu RT, Morris NR (1988). Mitotic induction and maintenance by overexpression of a G2-specific gene that encodes a potential protein kinase. Cell.

[21] Govindaraghavan M (2014). Identification of interphase functions for the NIMA kinase involving microtubules and the ESCRT pathway. PLoS Genet..

[22] Motose H, Tominaga R, Wada T, Sugiyama M, Watanabe Y (2008). A NIMA-related protein kinase suppresses ectopic outgrowth of epidermal cells through its kinase activity and the association with microtubules. Plant J..

[23] Sakai T (2008). Armadillo repeat-containing kinesins and a NIMA-related kinase are required for epidermal-cell morphogenesis in *Arabidopsis*. Plant J..

[24] Motose H (2011). NIMA-related kinases 6, 4, and 5 interact with each other to regulate microtubule organization during epidermal cell expansion in *Arabidopsis thaliana*. Plant J..

[25] Motose H, Takatani S, Ikeda T, Takahashi T (2012). NIMA-related kinases regulate directional cell growth and organ development through microtubule function in *Arabidopsis thaliana*. Plant Signal. Behav..

[26] Takatani S, Otani K, Kanazawa M, Takahashi T, Motose H (2015). Structure, function, and evolution of plant NIMA-related kinases: Implication for phosphorylation-dependent microtubule regulation. J. Plant Res..

[27] Vineyard L, Elliott A, Dhingra S, Lucas JR, Shaw SL (2013). Progressive transverse microtubule array organization in hormone-induced *Arabidopsis* hypocotyl cells. Plant Cell.

[28] Ambrose JC, Wasteneys GO (2008). CLASP modulates microtubule-cortex interaction during self-organization of acentrosomal microtubules. Mol. Biol. Cell.

[29] Zuo J, Niu QW, Chua NH (2000). An estrogen receptor-based transactivator XVE mediates highly inducible gene expression in transgenic plants. Plant J..

[30] Nogales E, Wolf SG, Downing KH (1998). Structure of the αβ-tubulin dimer by electron crystallography. Nature.

[31] Liu N (2015). Proteomic profiling and functional characterization of multiple post-translational modifications of tubulin. J. Proteome Res..

[32] Durek P (2010). PhosPhAt: the *Arabidopsis thaliana* phosphorylation site database. An update. Nuc, Acid. Res..

[33] Ishida T, Kaneko Y, Iwano M, Hashimoto T (2007). Helical microtubule arrays in a collection of twisting tubulin mutants of *Arabidopsis thaliana*. Proc. Natl. Acad. Sci. USA.

[34] Orbach MJ, Porro EB, Yanofsky C (1986). Cloning and characterization of the gene for β-tubulin from a benomyl-resistant mutant of *Neurospora crassa* and its use as a dominant selectable marker. Mol. Cell Biol..

[35] Jung MK, Wilder IB, Oakley BR (1992). Amino acid alterations in the *benA* (beta-tubulin) gene of *Aspergillus nidulans* that confer benomyl resistance. Cell Motil. Cytoskel..

[36] Li J, Katiyar SK, Edlind TD (1996). Site-directed mutagenesis of *Saccharomyces cerevisiae* β-tubulin: interaction between residue 167 and benzimidazole compounds. FEBS Lett..

[37] Hashimoto T (2002). Molecular genetic analysis of left-right handedness in plants. Philos. Trans. R. Soc. Lond. B Biol. Sci..

[38] Abe T, Hashimoto T (2005). Altered microtubule dynamics by expression of modified alpha-tubulin protein causes right-handed helical growth in transgenic *Arabidopsis* plants. Plant J..

[39] Oda Y, Iida Y, Kondo Y, Fukuda H (2010). Wood cell-wall structure requires local 2D-microtubule disassembly by a novel plasma membrane-anchored protein. Curr. Biol..

[40] Westermann S (2006). The Dam1 kinetochore ring complex moves processively on depolymerizing microtubule ends. Nature.

[41] Ambrose JC, Shoji T, Kotzer AM, Pighin JA, Wasteneys GO (2007). The *Arabidopsis* CLASP gene encodes a microtubule-associated protein involved in cell expansion and division. Plant Cell.

[42] Kirik V (2007). CLASP localizes in two discrete patterns on cortical microtubules and is required for cell morphogenesis and cell division in *Arabidopsis*. J Cell Sci..

[43] Eren EC, Gautam N, Dixit R (2012). Computer simulation and mathematical models of the noncentrosomal plant cortical microtubule cytoskeleton. Cytoskeleton.

[44] Hesse J, Thierauf M, Ponstingl H (1987). Tubulin sequence region β155-174 is involved in binding exchangeable guanosine triphosphate. J. Biol. Chem..

[45] Fourest-Lieuvin A (2006). Microtubule regulation in mitosis: tubulin phosphorylation by the cyclin-dependent kinase Cdk1. Mol. Biol. Cell.

[46] O’Regan L, Fry AM (2009). The Nek6 and Nek7 protein kinases are required for robust mitotic spindle formation and cytokinesis. Mol. Cell Biol..

[47] Verhey KJ, Gaertig J (2007). The tubulin code. Cell Cycle.

[48] Eng RC, Wasteneys GO (2014). The microtubule plus-end tracking protein Armadillo-Repeat Kinesin1 promotes microtubule catastrophe in *Arabidopsis*. Plant Cell.

[49] Nakamura M, Naoi K, Shoji T, Hashimoto T (2004). Low concentrations of propyzamide and oryzalin alter microtubule dynamics in *Arabidopsis* epidermal cells. Plant Cell Physiol..

[50] Takatani S, Hirayama T, Hashimoto T, Takahashi T, Motose H (2015). Abscisic acid induces ectopic outgrowth in epidermal cells through cortical microtubule reorganization in *Arabidopsis thaliana*. Sci Rep..

[51] Curtis MD, Grossniklaus U (2003). A gateway cloning vector set for high-throughput functional analysis of genes *in planta*. Plant Physiol..

[52] Nakagawa T (2007). Improved gateway binary vectors: high-performance vectors for creation of fusion constructs in transgenic analysis of plants. Biosci. Biotechnol. Biochem..

[53] Nakagawa T (2007). Development of series of gateway binary vectors, pGWBs, for realizing efficient construction of fusion genes for plant transformation. J. Biosci. Bioeng..

[54] Clough SJ, Bent AF (1998). Floral dip: a simplified method for *Agrobacterium*-mediated transformation of *Arabidopsis thaliana*. Plant J..

[55] Takahashi T, Naito S, Komeda Y (1992). Isolation and analysis of the expression of two genes for the 81-kilodalton heat-shock proteins from *Arabidopsis*. Plant Physiol..

[56] Ozawa S (2009). Biochemical and structural studies of the large Ycf4-photosystem I assembly complex of the green alga *Chlamydomonas reinhardtii*. Plant Cell.

