# Supplementary material for: Directional cell expansion requires NIMA-related kinase 6 (NEK6)-mediated cortical microtubule destabilization
Source: Sci Rep. 2017 Aug 10;7:7826. doi: 10.1038/s41598-017-08453-5 (PMC5552743; doi:10.1038/s41598-017-08453-5)
Supplement: Supplementary file 1 — Supplementary Information [file 41598_2017_8453_MOESM1_ESM.pdf]

SUPPLEMENTARY INFORMATION

Directional cell expansion requires NIMA-related kinase 6 (NEK6)-mediated cortical microtubule destabilization

Authors
Shogo Takatani[1], Shinichiro Ozawa[1,2], Noriyoshi Yagi[3], Takashi Hotta[3], Takashi Hashimoto[3], Yuichiro Takahashi[1,2], Taku Takahashi[1], Hiroyasu Motose[*]

[1] Department of Biological Science, Graduate School of Natural Science and Technology, Okayama University, 3-1-1 Tsushimanaka, Okayama 700-8530, Japan

[2] Japan Science and Technology Agency, 4-1-8 Kawaguchi, Saitama 332-0012, Japan

[3] Graduate School of Biological Science, Nara Institute of Science and Technology, Ikoma 630-0192, Japan

*Correspondence: Hiroyasu Motose (motose@cc.okayama-u.ac.jp)

Supplementary Figure S1

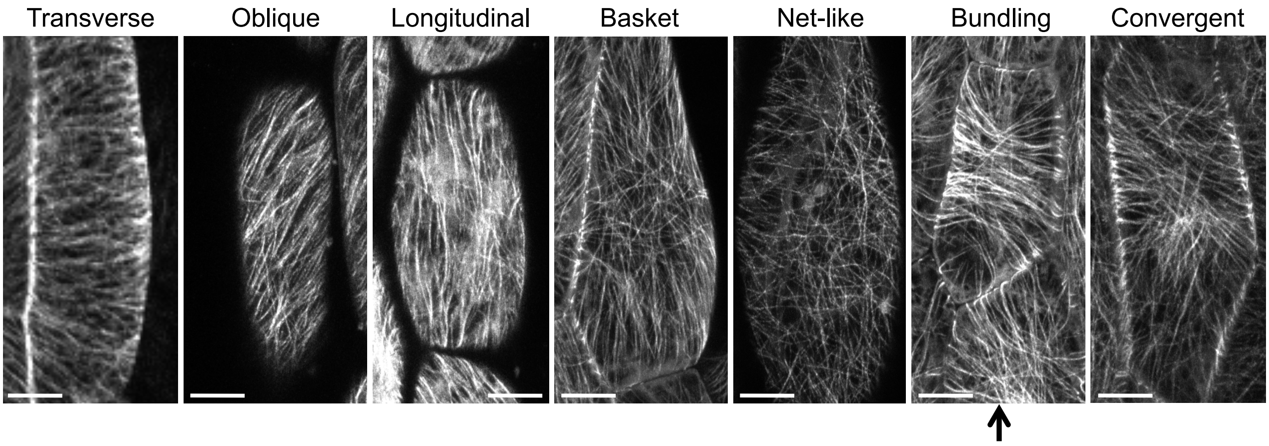

Supplementary Figure S1. Patterns of cortical microtubules. Cortical microtubules were observed in the epidermal cells of 3-day-old seedlings under a confocal microscopy. The image of bundling is identical to that of Figure 2e because it shows a most clear example of microtubule bundling.

Shogo Takatani, Shinichiro Ozawa, Noriyoshi Yagi, Takashi Hotta, Takashi Hashimoto, Yuichiro Takahashi, Taku Takahashi, Hiroyasu Motose

Directional cell expansion requires NIMA-related kinase 6 (NEK6)-mediated cortical microtubule destabilization

Supplementary Figure S2

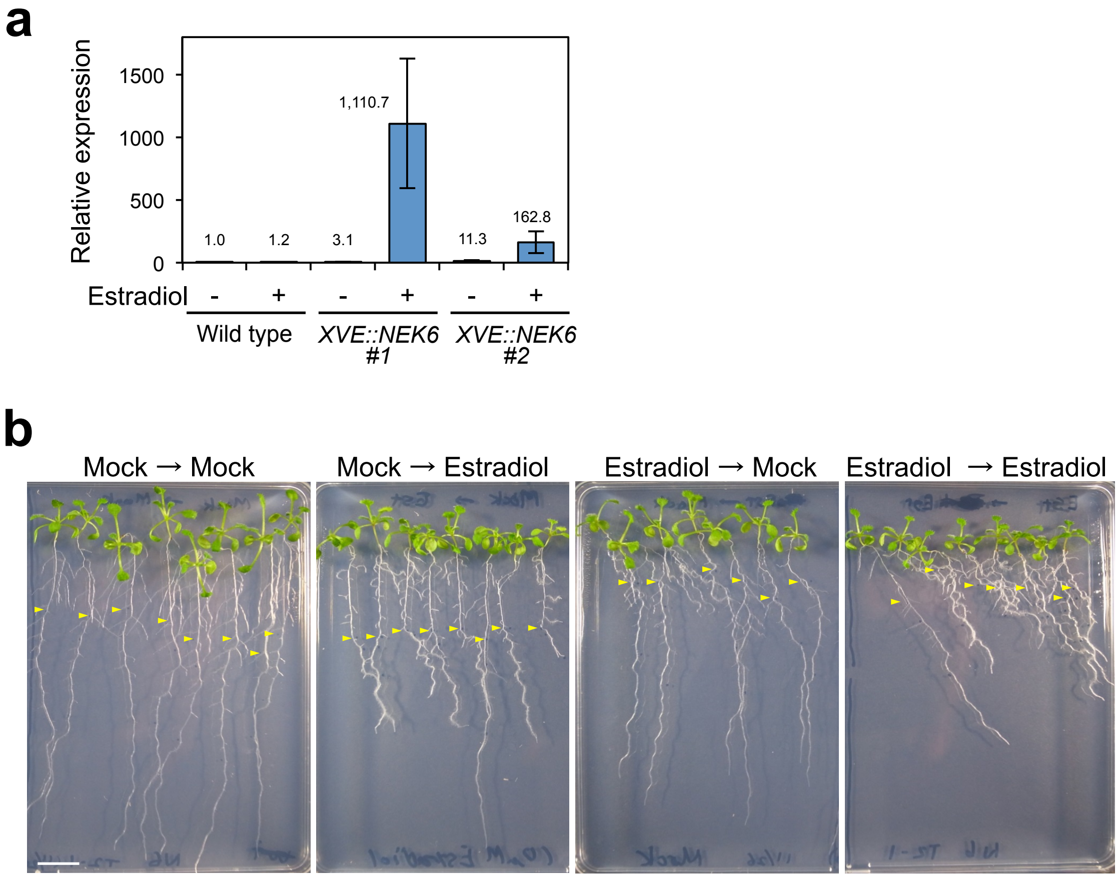

Supplementary Figure S2. *NEK6* induction and its reversible effect on root growth. **a**, Accumulation of *NEK6* transcripts in the wild type (WT) or two independent transgenic lines harboring estradiol-inducible *NEK6* construct (*XVE::NEK6* #1 and #2) grown for 7 days in the absence (-) or presence (+) of estradiol. All transcript levels are relative to that of mock-treated wild type plants. Data are displayed as averages ± SEM (n = 3 independent experiments). **b**, Reversible effect of estradiol-inducible *NEK6* overexpression. The seedlings harboring estradiol-inducible *NEK6* construct (*XVE::NEK6* #1) were grown for 7 days in the absence (Mock) or presence (Estradiol) of estradiol and then transferred to medium with or without estradiol. After transfer, seedlings were grown for 7 days. Arrowheads indicate the position of the root apex at the time of transfer to new medium. The scale bar represents 1 cm.

Shogo Takatani, Shinichiro Ozawa, Noriyoshi Yagi, Takashi Hotta, Takashi Hashimoto, Yuichiro Takahashi, Taku Takahashi, Hiroyasu Motose

Directional cell expansion requires NIMA-related kinase 6 (NEK6)-mediated cortical microtubule destabilization

Supplementary Figure S3

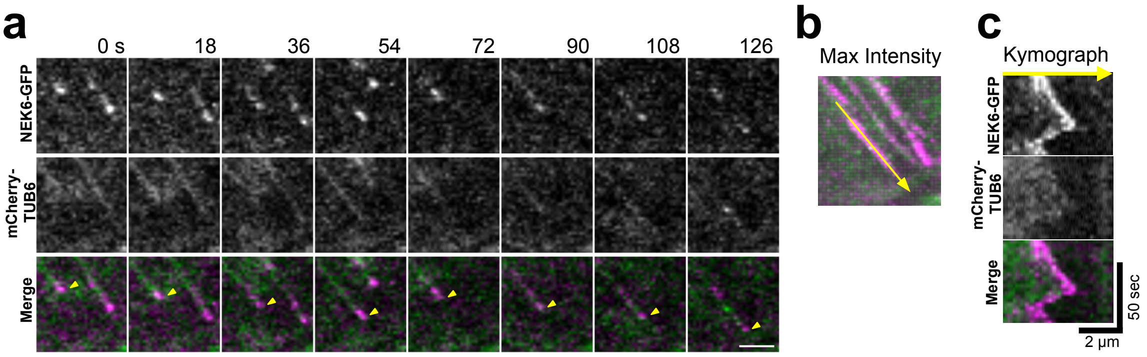

Supplementary Figure S3. Localization of NEK6-GFP to both shrinking and growing ends in the basal region of hypocotyls. **a**, A time-lapse montage showing that NEK6-GFP localizes to growing and shrinking plus ends. Arrowheads indicate the plus end of a microtubule. The scale bar represents 2 µm. **b**, A maximum intensity projection of **(a)**. The yellow line shows the location of the kymograph line. **c**, Kymograph of the line in **(b)**.

Shogo Takatani, Shinichiro Ozawa, Noriyoshi Yagi, Takashi Hotta, Takashi Hashimoto, Yuichiro Takahashi, Taku Takahashi, Hiroyasu Motose

Directional cell expansion requires NIMA-related kinase 6 (NEK6)-mediated cortical microtubule destabilization

Supplementary Figure S4

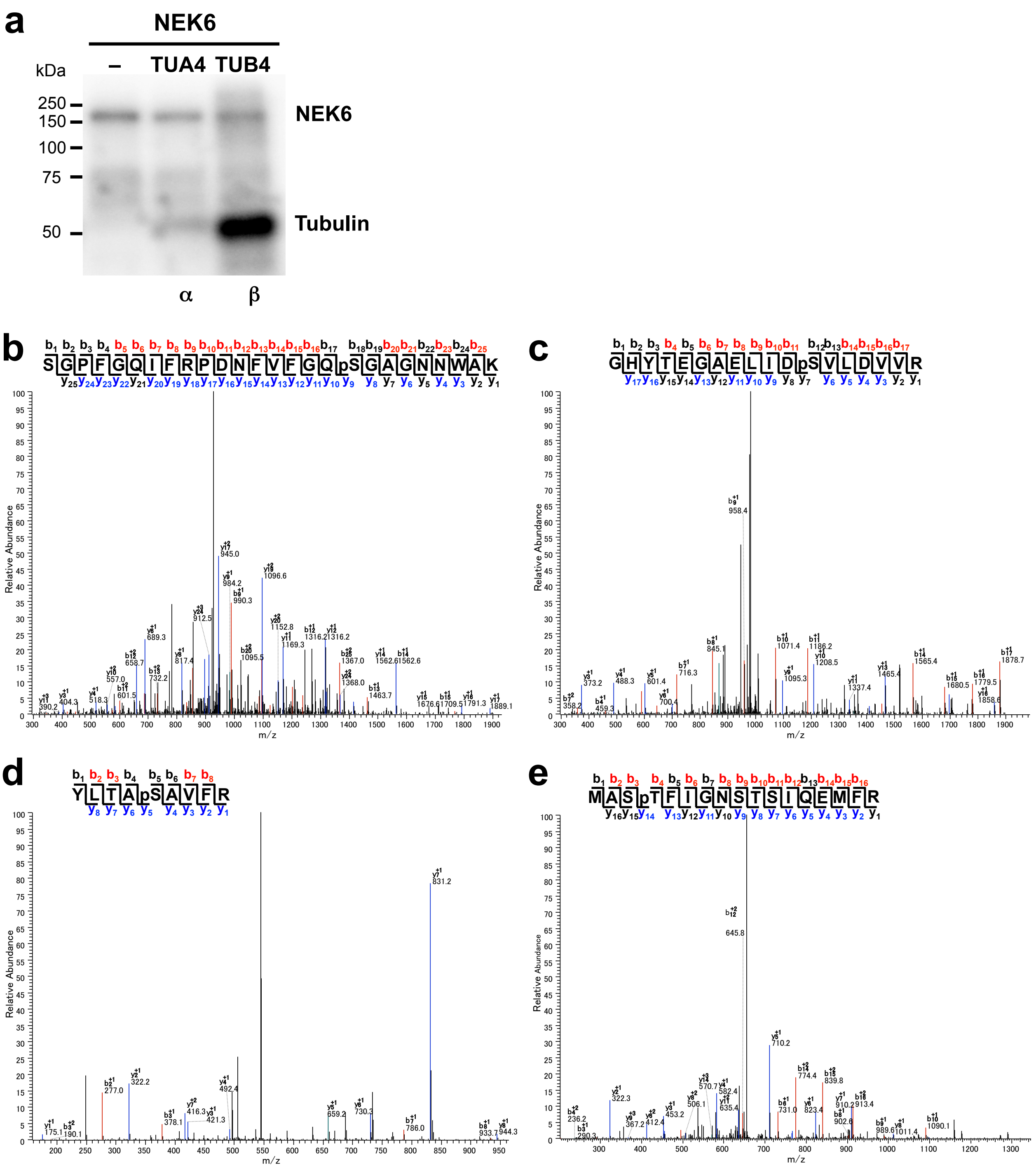

Supplementary Figure S4. NEK6 phosphorylation of β-tubulin. **a**, NEK6 phosphorylates β-tubulin but not α-tubulin. Recombinant 6xHis-TUB4 or 6xHis-TUA4 was incubated with GST-NEK6 in the presence of [γ-^{32}P]ATP, separated by SDS-PAGE, and analyzed by autoradiography. **b-e**, MS/MS spectra of four phosphopeptides from NEK6-treated TUB4. **b**, Ser-95 (78-SGPFGQIFRPDNFVFGQpSGAGNNWAK-103). **c**, Ser-115 (104-GHYTEGAELIDpSVLDVVR-121). **d**, Ser-314 (310-YLTApSAVFR-318). **e**, Thr-366 (363-MASpTFIGNSTSIQEMFR-379).

Shogo Takatani, Shinichiro Ozawa, Noriyoshi Yagi, Takashi Hotta, Takashi Hashimoto, Yuichiro Takahashi, Taku Takahashi, Hiroyasu Motose
Directional cell expansion requires NIMA-related kinase 6 (NEK6)-mediated cortical microtubule destabilization

Supplementary Figure S5

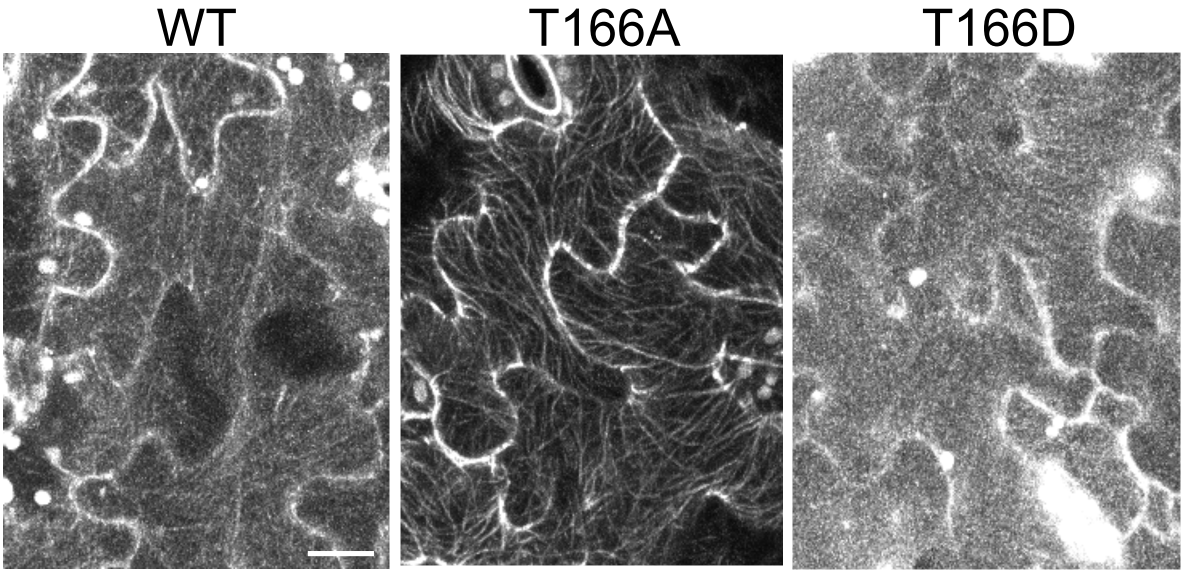

Supplementary Figure S5. Subcellular localization of wild type TUB4 (WT) and mutated TUB4 proteins (T166A and T166D) fused with GFP at the N-terminus in cotyledon epidermal cells. The scale bar represent 10 μm.

Shogo Takatani, Shinichiro Ozawa, Noriyoshi Yagi, Takashi Hotta, Takashi Hashimoto, Yuichiro Takahashi, Taku Takahashi, Hiroyasu Motose

Directional cell expansion requires NIMA-related kinase 6 (NEK6)-mediated cortical microtubule destabilization

Supplementary Figure S6

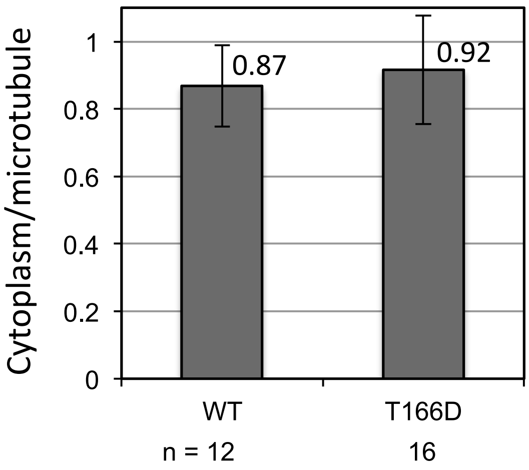

Supplementary Figure S6. The ratio of fluorescent signal intensity of cytoplasmic free GFP-TUB4 to that of microtubule-localized GFP-TUB4. The fluorescent signal of microtubule-localized GFP-TUB4 was quantified in the cortical focal planes. The fluorescent signal of cytoplasmic free GFP-TUB4 was quantified in the subcortical cytoplasmic focal planes. Data are displayed as averages ± SDs. The averages are shown in the graph.

Shogo Takatani, Shinichiro Ozawa, Noriyoshi Yagi, Takashi Hotta, Takashi Hashimoto, Yuichiro Takahashi, Taku Takahashi, Hiroyasu Motose

Directional cell expansion requires NIMA-related kinase 6 (NEK6)-mediated cortical microtubule destabilization

Supplementary Figure S7

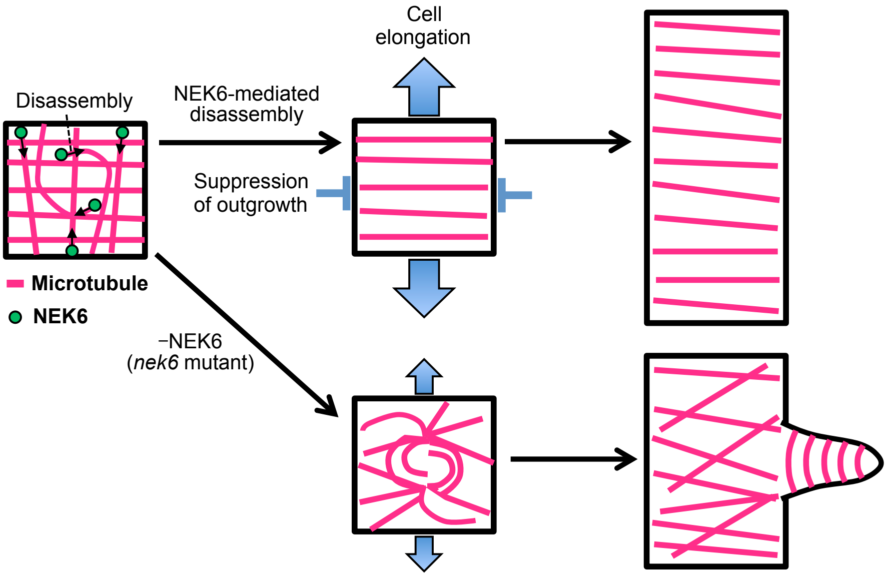

Supplementary Figure S7. Schematic model of NEK6 function in directional growth. NEK6 phosphorylates β-Tubulin and destabilizes cortical microtubules. NEK6-mediated microtubule disassembly eliminates aberrant microtubules both to promote cell elongation and to suppress ectopic outgrowth.

Shogo Takatani, Shinichiro Ozawa, Noriyoshi Yagi, Takashi Hotta, Takashi Hashimoto, Yuichiro Takahashi, Taku Takahashi, Hiroyasu Motose

Directional cell expansion requires NIMA-related kinase 6 (NEK6)-mediated cortical microtubule destabilization

Supplementary Table S1. Catastrophe frequency of detached microtubules.

Parameters	WT	*nek6-1*
Number of catastrophe events	67	48
Total time of growth (sec)	3049	4663
Catastrophe frequency (event sec^{-1})	0.022	0.010

The catastrophe frequency was calculated by dividing the number of catastrophe events by the total time of growth.

Supplementary Table S2. Parameters of microtubule dynamic instability.

Dynamic parameters	WT	*nek6-1*
Growing rate (µm min^{-1})	8.02±1.57	7.59±1.60
Shrinking rate (µm min^{-1})	21.70±7.30	19.57±5.50
Catastrophe frequency (event sec^{-1})	0.018	0.019
Rescue frequency (event sec^{-1})	0.031	0.030
Time spent, %		
Growth	70.58±8.01	68.30±4.92
Pause	22.33±8.21	21.48±3.73
Shrinkage	7.09±5.00	10.23±7.13
Dynamicity (µm min^{-1})	10.28±1.38	9.94±2.82

The catastrophe frequency was calculated by inversing the mean time spent in growth and the rescue frequency was calculated by inversing the mean time spent in shrinkage. Dynamicity was calculated by dividing the sum of the length of growth and shrinkage by the total time a particular microtubule was observed. Values are means ± SDs. Sample values were: wild type (WT), n = 56 microtubules and t = 5385 seconds; nek6-1, n = 56 microtubules and t = 5509 seconds.

Supplementary Table S3. Primers Used in This Study.

**TUB4 site-directed mutagenesis**	
TUB4-F-S95A	gCTGGTGCCGGAAATAACTGGGC
TUB4-F-S95D	gaTGGTGCCGGAAATAACTGGGC
TUB4-R-Q94	TTGACCAAAGACGAAGTTATCAGGA
TUB4-F-S115A	gCTGTTCTCGATGTTGTGAGGAAG
TUB4-F-S115D	gaTGTTCTCGATGTTGTGAGGAAG
TUB4-R-D114	ATCAATCAACTCAGCACCTTCGGT
TUB4-F-T166A	gCTTTCTCAGTGTTTCCTTCTCCTA
TUB4-F-T166D	gaTTTCTCAGTGTTTCCTTCTCCTA
TUB4-R-M165	CATCATCATACGATCTGGATACTCT
TUB4-F-S314A	gCCGCTGTGTTCCGTGGAAAGCTG
TUB4-F-S314D	gaCGCTGTGTTCCGTGGAAAGCTG
TUB4-R-A313	TGCAGTCAAGTAACGTCCATGACG
TUB4-F-T366A	gCTTTCATTGGTAACTCAACCTCAATC
TUB4-F-T366D	gaTTTCATTGGTAACTCAACCTCAATC
TUB4-R-S365	AGACGCCATTTTCAAACCCTTTGGT
**NEK6 site-directed mutagenesis**	
IBO1-Fm-E177R(1-1)	aAACTGCTTGCTGATATCCCTTATGGTT
IBO1-R-P176	CGGGCACATGTAGTTTGGAGTTCC
**TUB4 promoter**	
TUB4pro-F-HindIII	aaaaAAGCTTTTGATACTCTTCAAAACCATCAAACCATGT
TUB4pro-R-XbaI	aaaaTCTAGATTTTTTTTTTTTTTGGTTTCTTCGTCAAGAG
**RT-qPCR**	
NEK6-RT-F	CCGGAGATCTGCTCATCAAGAGG
NEK6-RT-R	GGAGAAGAGTCAATCACTGGCATATGAT
ACTIN8-RT-F	GTGAGCCAGATCTTCATTCGTC
ACTIN8-RT-R	TCTCTTGCTCGTAGTCGACAG

Supplementary Table S4. TUB4 Peptides Detected by LC-MS/MS.

Peptide	Position	Z	DeltaM	Xcorr
EILHIQGGQCGNQIGAK	3 - 19	1	-0.21	4.3
EILHIQGGQCGNQIGAK	3 - 19	2	0.25	4.4
EILHIQGGQCGNQIGAK	3 - 19	3	-0.30	4.2
FWEVICDEHGIDHTGQYVGDSPLQLER	20 - 46	2	0.15	5.1
FWEVICDEHGIDHTGQYVGDSPLQLER	20 - 46	3	0.07	7.4
IDVYFNEASGGK	47 - 58	2	0.38	4.1
IDVYFNEASGGKYVPR	47 - 62	2	-0.25	4.5
IDVYFNEASGGKYVPR	47 - 62	3	0.57	3.1
AVLMDLEPGTMDSLR	63 - 77	1	-0.23	2.5
AVLMDLEPGTMDSLR	63 - 77	2	0.16	3.4
SGPFGQIFRPDNFVFGQSGAGNNWAK	78 - 103	2	0.01	5.3
SGPFGQIFRPDNFVFGQSGAGNNWAK	78 - 103	3	0.42	7.7
SGPFGQIFRPDNFVFGQS*GAGNNWAK	78 - 103	3	0.06	7.6
PDNFVFGQSGAGNNWAK	87 - 103	2	0.33	4.9
PDNFVFGQSGAGNNWAK	87 - 103	3	0.84	4.8
GHYTEGAELIDSVLDVVR	104 - 121	1	0.88	4.2
GHYTEGAELIDSVLDVVR	104 - 121	2	0.16	6.8
GHYTEGAELIDSVLDVVR	104 - 121	3	0.40	2.6
GHYTEGAELIDS*VLDVVR	104 - 121	2	0.00	7.4
GHYTEGAELIDS*VLDVVR	104 - 121	3	0.87	3.2
GHYTEGAELIDSVLDVVRK	104 - 122	3	1.29	5.0
EAENSDCLQGFQVCHSLGGGTGSGMGTLLISK	123 - 154	3	0.48	5.1
KEAENSDCLQGFQVCHSLGGGTGSGMGTLLISK	122 - 154	2	-1.98	3.1
KEAENSDCLQGFQVCHSLGGGTGSGMGTLLISK	122 - 154	3	0.50	5.1
IREEYPDR	155 - 162	2	0.13	2.8
MMMTFSVFPSPK	163 - 174	2	0.15	4.1
MMMT*FSVFPSPK	163 - 174	2	0.28	4.5
LANPTFGDLNHLISATMSGVTCCLR	217 - 241	2	0.02	4.4
LANPTFGDLNHLISATMSGVTCCLR	217 - 241	3	0.51	6.4
FPGQLNSDLR	242 - 251	1	-0.18	2.5
FPGQLNSDLR	242 - 251	2	-0.08	3.1

FPGQLNSDLRK	242 - 252	2	-0.15	2.7
R.KLAVNLIPFPR.L	252 - 262	1	-0.22	2.6
KLAVNLIPFPR	252 - 262	2	-0.21	3.5
LAVNLIPFPR	253 - 262	2	-0.06	3.3
LAVNLIPFPRLHFFMVGFAPLTSR	253 - 276	3	0.68	5.1
LHFFMVGFAPLTSR	263 - 276	1	-0.25	3.8
LHFFMVGFAPLTSR	263 - 276	2	0.23	4.5
LHFFMVGFAPLTSR	263 - 276	3	0.43	4.0
GSQQYSALSVPELTQQMWDAK	277 - 297	2	0.03	4.3
GSQQYSALSVPELTQQMWDAK	277 - 297	3	0.64	4.4
NMMCAADPR	298 - 306	2	-0.14	2.8
YLTASAVFR	310 - 318	2	0.51	2.9
YLTAS*AVFR	310 - 318	2	0.37	3.5
LSTKEVDEQMMNIQNK	321 - 336	3	1.45	4.2
EVDEQMMNIQNK	325 - 336	1	-0.12	3.3
EVDEQMMNIQNK	325 - 336	2	0.38	4.0
EVDEQMMNIQNKNSSYFVEWIPNNVK	325 - 350	3	1.35	3.8
NSSYFVEWIPNNVK	337 - 350	1	0.63	3.3
NSSYFVEWIPNNVK	337 - 350	2	0.12	3.8
NSSYFVEWIPNNVK	337 - 350	3	1.23	3.0
SSVCDIAPK	351 - 359	2	-0.03	2.7
GLKMASTFIGNSTSIQEMFR	360 - 379	3	0.72	5.2
MASTFIGNSTSIQEMFR	363 - 379	1	0.71	3.3
MASTFIGNSTSIQEMFR	363 - 379	2	-0.09	5.1
MASTFIGNSTSIQEMFR	363 - 379	3	0.04	4.1
MAST*FIGNSTSIQEMFR	363 - 379	2	0.13	4.3
MAST*FIGNSTSIQEMFR	363 - 379	3	1.81	4.7
RVSEQFTAMFR	380 - 390	2	-0.42	3.5
RVSEQFTAMFR	380 - 390	3	0.68	2.9
VSEQFTAMFR	381 - 390	2	0.15	3.5

The charge state (Z) of the measured ion, the calculated deviation (DeltaM), and the cross correlation factor (Xcorr) caluculated by the Sequest algorithm are given. The position of the peptide within the protein sequence is also listed. Asterisks indicate phosphorylated residues (highlighted in red).

Supplementary Movie legends

Supplementary Movie S1. Cortical microtubule dynamics in a hypocotyl epidermal cell of the wild type. Images were acquired every 3 seconds.

Supplementary Movie S2. Cortical microtubule dynamics in a hypocotyl epidermal cell of *nek6-1* before ectopic outgrowth. Images were acquired every 3 seconds.

Supplementary Movie S3. Cortical microtubule dynamics in a hypocotyl epidermal cell of *nek6-1* before ectopic outgrowth. A close-up view of Movie S2 (four repeats of a microtubule-bending event). Images were acquired every 3 seconds. Movie corresponds to Figure 3a.

Supplementary Movie S4. Detached and distorted cortical microtubule in a hypocotyl epidermal cell of *nek6-1*. Circle marks microtubule plus end. Images were acquired every 3 seconds. Movie corresponds to Figure 3b.

Supplementary Movie S5. Detached and distorted cortical microtubule in a cotyledon epidermal cell of *nek6-1*. Circle marks microtubule plus end. Images were acquired every 2 seconds. Movie corresponds to Figure 3c.

Supplementary Movie S6. NEK6 localizes to both plus and minus shrinking ends of microtubules. Circles and squares mark plus and minus shrinking ends, respectively. Images were acquired every 2 seconds. Movie corresponds to Figure 5a and 5d.

Supplementary Movie S7. NEK6 does not localize to the growing ends of microtubules. Circles mark growing microtubule ends. Images were acquired every 2 seconds. Movie corresponds to Figure 5g.

Supplementary Movie S8. NEK6 localizes to both growing and shrinking microtubule ends in the basal region of hypocotyls. Images were acquired every 3 seconds. Movie corresponds to Supplementary Figure 3a.

Supplementary Movie S9. NEK6 localizes to the shrinking end of a detached and distorted cortical microtubule. The yellow arrowhead indicates the NEK6-GFP-localized shrinking end. The white line indicates the detached microtubule. Images were acquired every 3 seconds. Movie corresponds to Figure 5l.

Supplementary Movie S10. NEK6 localizes to the shrinking end of a detached and distorted cortical microtubule. The yellow arrowhead indicates the NEK6-GFP-localized shrinking end. The white line indicates the detached microtubule. Images were acquired every 3 seconds. Movie corresponds to Figure 5m.

Supplementary Movie S11. Visualization of microtubules in root meristematic zone by TUB4pro:GFP-TUB4^{T166A}. A set of 31 optical sections at 0.65 μm z-spacing.

Supplementary Movie S12. Visualization of microtubules in root elongation zone by TUB4pro:GFP-TUB4^{T166A}. A set of 31 optical sections at 0.5 μm z-spacing.
